# A reference-free pipeline for detecting shared transposable elements from pan-genomes to retrace their dynamics in a species

**DOI:** 10.1186/s13059-026-03984-5

**Published:** 2026-02-07

**Authors:** Somia Saidi, Mathieu Blaison, María del Pilar Rodríguez-Ordóñez, Johann Confais, Hadi Quesneville

**Affiliations:** Université Paris-Saclay, INRAE, BioinfOmics, URGI, Versailles, 78026 France

## Abstract

**Background:**

The role of transposable elements (TEs) in host adaptation has gained interest in recent years. Individuals of the same species undergo independent TE insertions, providing genetic variability within populations, upon which natural selection can act to foster adaptation to environmental conditions.

**Results:**

As de novo assembled genomes are becoming increasingly affordable, helping to overcome the bias introduced by relying on a single reference genome, there is a growing need for suitable pangenomic tools to explore the genomic diversity within a species. We developed a new pipeline called panREPET that identifies TE insertions shared by groups of individuals. Unlike other pangenomic tools, panREPET operates independently of a reference genome and provides the precise sequence and genomic coordinates of each TE copy for each genome.

**Conclusions:**

We showcase the potential of this tool by identifying TE insertions shared among 42 *Brachypodium distachyo*n genomes and by comparing our results with those of existing tools to demonstrate its advantages. Using panREPET, we were able to date two major TE bursts corresponding to major climate events: 22 kya during the Last Glacial Maximum and 10 kya during the Holocene, showing a potential link between environmental stress and TE activity.

**Supplementary Information:**

The online version contains supplementary material available at 10.1186/s13059-026-03984-5.

## Introduction

Transposable elements (TEs) are mobile DNA sequences that can invade genomes through transposition. Their insertions may be neutral for the host or deleterious, for instance, by disrupting gene expression [1–3]. Several mechanisms may eliminate them, such as host defence [1] or purifying selection [4]. TE insertions may also be beneficial in fostering genome evolution through introduction of functional novelties [5]. In natural populations, TE insertion polymorphism frequency is subject to drift, selection and population migrations [6]. Understanding how TEs can enable a species to adapt to a local environment requires a detailed study of their insertions in the genomes of different individuals. At the species level, a pangenomic approach seeks to detect intraspecific TE diversity and to reconstruct their dynamics. A TE pangenome can be defined by (i) TE insertions present in all individuals of the species (core genome), (ii) insertions present in a subset of individuals, and (iii) individual-specific TE insertions. Some TEs are known to respond to abiotic stress and can therefore modulate the expression of nearby genes under changing environmental conditions [5, 7, 8]. Using pangenomic approaches, several studies have shown that recent intraspecific TE insertions can contribute to local adaptation. In *Arabidopsis thaliana*, a population genomic analysis revealed extensive variation in ATCOPIA78 copy number that correlates with temperature variation [9]. In rice, TE activation was suggested to be triggered by external stimuli rather than by defects in TE silencing pathways [10].

Detecting TE polymorphisms is challenging not only due to their repetitive nature, but also because insertions absent from the reference genome cannot be detected when reads from multiple genomes are aligned to it. In addition, regions absent from the reference genome are unavailable for analysis as they are not assembled. However, multiple de novo assembled genomes for the same species become available. We therefore developed a new pipeline, called panREPET, that does not depend on a reference genome to study TE insertions in a species. panREPET overcomes the reference dependency by comparing TE copies between each pair of de novo assembled genomes. panREPET can also facilitate dating of TE dynamics at the species level for all types of TE sequences by integrating SNP-based genetic distances between pairwise accessions.

To showcase the potential of panREPET, we tested it on *Brachypodium distachyon*, a species for which many genome sequences are available. This annual Mediterranean grass, which has emerged as a genomic model, remains undomesticated, offering an opportunity to investigate TE dynamics in the context of adaptation to the mosaic of Mediterranean climates [11]. Here, we used 54 de novo whole-genome assemblies available for *B. distachyon*, of which 42 were retained after filtering for assembly quality [12]. These accessions, initially sequenced to establish the *B. distachyon* pangenome, occur in contrasting ecological niches, are genetically diverse, and represent three (B_West, B_East, and A_East) of the five genetic clades identified in this species [13]. In contrast to more recently sequenced accessions from Italy and the Balkans [13, 14], the high coverage employed by Gordon et al*.* [12, 14] (median genome coverage of 92x; mean assembled genome size of 268 Mbp, close to the 272 Mbp reference) enabled de novo assemblies. Because each accession has been annotated for genes and used to investigate TE dynamics with short-read and population genomic approaches [12–15], they constitute an ideal dataset to test and benchmark panREPET. To illustrate the potential of panREPET, we address the following questions about *B. distachyon* TEs: What are the bursts of transposition in this species? Which TE families were involved, and when did these events occur? For the oldest insertions, what mechanisms explain their conservation? And when a recent increase in transposition is observed in only a few individuals, might external biotic or abiotic factors have triggered this activation?

Here, we first present the panREPET pipeline and compare its results to those of alternative tools used in recent TE pangenomic studies to demonstrate its performance. In a second part, we illustrate its application to 42 genomes from *B. distachyon*. We describe the TE evolutionary history by estimating transposition burst events. We found a co-occurrence between the dates of these events and past climate changes, suggesting that climate factors may, in some cases, explain TE dynamics.

## Results

### panTEannot: a TEannot adaptation

**Fig. 1:**
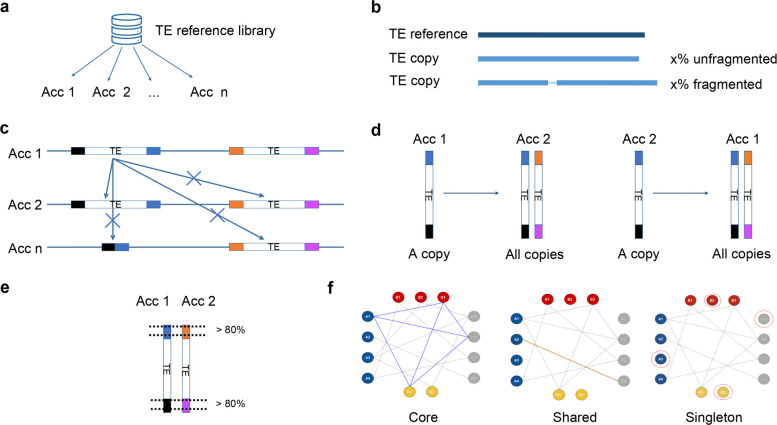
panREPET steps. **a** Independent TE genome annotation with panTEannot using a TE reference library. **b** Extraction of copies according to their coverage percentage in the alignment with their reference (e.g., *x* is between 95 and 105% for complete copies). **c** A TE copy from accession 1 is matched to TE copies with the same flanking regions to detect copies located at the same genomic locus. **d** Bidirectional best-hit detection. **e** A minimum of 80% overlap between the flanking regions of the two matched TE copies is required to validate their alignment. **f** Clique extraction to identify TE insertions. Copy classification: core, shared, or singleton. A circle represents a TE copy, a color represents an accession, and a bold line represents a bidirectional best-hit detection. Acc: accession

The TE reference library was built from the Bd21 v3.2 genome sequence using the TEdenovo pipeline of the REPET package [16] (see Methods, TE library building). Our pangenomic approach aims to capture as much diversity as possible across genomes. Consequently, we independently annotated each genome for its TE content using TEannot with the Bd21 TE reference library (Fig. 1a). However, this TE annotation process is time-consuming and requires a faster way to annotate the 54 genomes (Table 1). Accordingly, we modified the TEannot pipeline from the REPET package [17] to produce a lighter version. Specifically, because we are interested in inter-individual TE variability, which is mainly driven by recent transposition events, we focused on non-degenerate sequences, i.e., TE copies that are complete relative to their reference sequence. Our goal is to identify Full-Length Fragment Copies (FLFs) and Full-Length Copies (FLCs) that align at least 95% of their consensus sequence [18]. FLFs are unfragmented copies, while FLCs may have gaps due to insertions but still align to more than 95% of the consensus. In the TEannot pipeline of REPET, the similarity search step is performed by Blaster [19], Censor [20], and RepeatMasker [21] to maximize sensitivity. Because complete TE copies are easy to detect, there is no need for such high sensitivity. We therefore retained only Blaster and removed Censor and RepeatMasker. Using the same rationale, we also removed SSR search, spurious hit removal, and fragment connection (TEannot steps 4, 5, and 7), as they have low impact on the detection of complete TEs. To improve parallel computation, we implemented it using the Snakemake framework [22], removing MySQL dependencies and avoiding the need for a cluster job scheduler such as Sun Grid Engine or Slurm. This new implementation is called panTEannot. As we aim to maintain good sensitivity and specificity with a faster method, we benchmarked its performance by comparing panTEannot to TEannot from REPET v3.0. We performed a complete TE annotation with TEannot from REPET v3.0 on the Bd21 reference genome sequence to be used as the gold standard. We also ran TEannot from REPET v3.0 without Censor and RepeatMasker to emulate panTEannot. We compared the different TE annotations against the gold standard at the nucleotide level (Table 1). We calculated sensitivity and specificity as in Baud et al*.* [23] (see Methods, panTEannot performance analysis). As expected, TEannot from REPET 3.0 has higher sensitivity (0.9985) than panTEannot (0.8951). However, panREPET shows higher specificity (0.9267 vs 0.8979) and is faster. In a pangenomic context, we are more interested in detecting true positives than in recovering every possible copy, including very old and degenerate ones, so we considered this a satisfactory compromise.

**Table 1:** Comparison of the performance of TEannot from REPET 3.0 and panTEannot on the Bd21 v3.2 reference genome. Real times were obtained on a 32-core, 64-GB RAM virtual machine

Annotation type	TE coverage	Sensitivity	TE intersect	Specificity	Gene intersect	Accuracy	Real time
**Gold standard:** **TEannot from REPET** Blaster + Censor + RepeatMasker	0.3705	0.9985	1	0.8979	0.1550	0.9468	19:25:40
**TEannot from REPET** Blaster + RepeatMasker	0.3583	0.9629	0.8799	0.9117	0.1199	0.9366	15:22:13
**TEannot from REPET** Blaster	0.3389	0.9092	0.7267	0.9245	0.1001	0.9170	12:50:55
**panTEannot** Blaster + RepeatMasker	0.3550	0.9459	0.8994	0.9126	0.1332	0.9288	00:39:46
**panTEannot** Blaster	0.3334	0.8951	0.7127	0.9267	0.0984	0.9114	00:20:02

Across the 54 genomes, panTEannot completed in 48 h on a 32-core, 64-GB RAM virtual machine, which we consider to be good performance. Mean TE sequence coverage is 29.9%. Missing genomic regions in genome assemblies are mainly due to repetitive content, including TEs. We observed that TE coverage depends on assembly quality: genomes with TE coverage below 30% have an assembled genome size (after removing “N” bases) that is less than 95% of the 256 Mbp reference genome (Additional file 2: Table S1). Using this threshold, we excluded ABR8, Bd3_1, Bd30-1, BdTR11a, BdTR12c, BdTR5i, BdTR8i, Gaz-8, Koz_3, Mur1, Tek-2, and Tek-4 from our study. Notably, the BdTR11a genome is larger than 256 Mbp (about 266 Mbp) but shows a TE sequence coverage of only 19%. Another useful metric for evaluating TE detection quality is the contig N50: the longer the contigs, the better the detection of TE copies. Consequently, we removed BdTR11a because, in addition to having a small assembled genome size, its contig N50 is low compared to the others (17 kbp versus a median of 24 kbp).

### panREPET pipeline

We developed the panREPET pipeline to identify shared TE insertions between genomes by comparing them pairwise. We divided the pipeline into the following steps:

- Step 1: Extract full-length TE copies based on reference alignment coverage
  The coverage percentage between a TE copy and its reference sequence from the TE library (the *covcons* parameter) is calculated as the length of the aligned region (in base pairs) divided by the total length of the reference sequence, multiplied by 100. This parameter enables the extraction of either complete or partial TE copies relative to their reference (Fig. 1b). By default, we only extract copies covering 95–105% of their consensus, which allows the selection of copies with relatively small sequence divergence from the reference. Values above 100% may occur when a copy is longer than its consensus, for example due to an insertion [18]. Limiting the extraction of TE copies by setting the *covcons* threshold also reduces computational time, particularly for large genomes.
- Step 2: Add flanking genomic sequence
  In order to recognize copies that are located at the same genomic locus, we retrieve their flanking sequences by simple coordinate extension on both sides using *bedtools slop* from the Bedtools package [24] (Fig. 1c). By default, we set the extension length to 500 bp, as it seemed the best compromise between detection performance and sensitivity in analysis of eight *Arabidopsis thaliana* genomes (see Additional file 6: Supplementary Results, detailed panREPET pipeline). This parameter can be modified by the user.
- Step 3: Bidirectional best-hit detection
  Bidirectional best-hit detection first begins with aligning TE copies extended by their flanking sequences between accessions to find their best hits and vice versa (Fig. 1d). Only pairs of reciprocal best hits are retained. This approach was inspired by methods for orthologous gene detection [25]. Pairwise comparisons are performed by Minimap2 [26], followed by Matcher [19], which merges nearby collinear alignment fragments. Furthermore, panREPET offers the possibility of comparing only TE copies present on the same chromosomes between accessions, which improves computational efficiency and reduces false positives by avoiding matches across unrelated chromosomes. However, this setting may miss TE copies relocated by translocations or segmental duplications and is therefore adjustable by the user depending on the biological context. It is then possible to highlight shared TE insertions that have undergone an inter-chromosomal translocation event or cases of segmental duplication corresponding to paralog copies (see Discussion, Potential applications). Homologous TE copies between two individuals are identified based on internal insertions or deletions within the TE sequence itself, rather than differences in the flanking regions. Indeed, to avoid false positives, we added a filtering step requiring that the two copies from the two individuals overlap by at least 80% in their flanking regions (Fig. 1e).
- Step 4: From bidirectional best hits to TE insertions
  Pairs of TE copies identified in step 3 are used to construct an undirected graph in which nodes represent TE copies and edges connect copies identified as reciprocal best hits. In graph theory, a clique is a subset of nodes in which every node is connected to every other node. We consider a clique as a proxy for a unique TE insertion found at the same locus in several genomes. We identified cliques using NetworkX v2.4 [27]. First, graphs were built from the pairs of reciprocal best hits using the *read_edgelist* function. Then, maximal cliques were detected with the *find_cliques* function. For any given node *n*, a maximal clique containing *n* is a complete subgraph that cannot be expanded by adding an adjacent node. A locus may belong to several cliques if the corresponding TE copy has diverged sufficiently between at least two groups of individuals.
  Finally, each TE copy in each genome is classified as core, shared, or singleton if it is found in all genomes, in a subset of genomes, or in only one genome, respectively (Fig. 1f). Adding or removing a genome does not require restarting the pipeline from the beginning, since pairwise genome comparisons are independent of the clique-building step.

### Retracing shared TE insertions

**Fig. 2:**
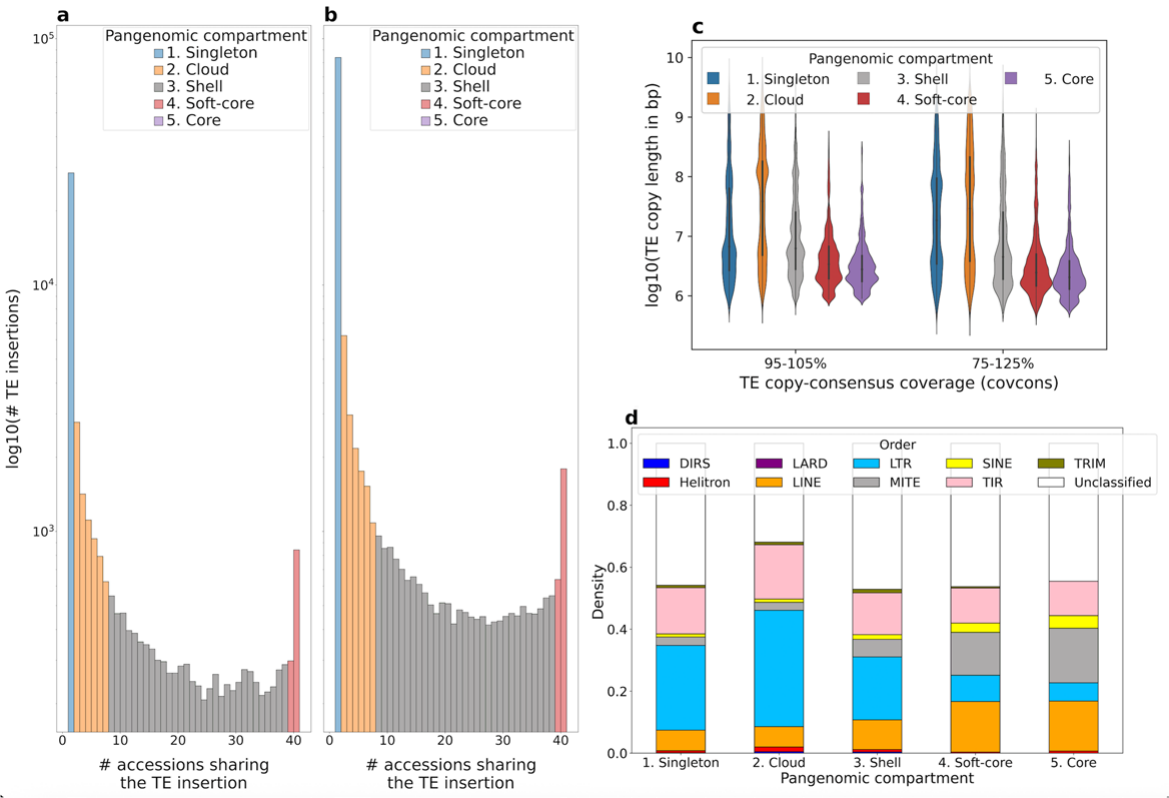
**a**, **b** Histograms showing the log_10_-transformed count of TE insertions across accessions, stratified by the percent coverage of TE copies relative to their consensus (*covcons*). **a** *covcons* = 95–105% **b** *covcons* = 75–125% **c** Distribution of TE copy lengths in base pairs across pangenomic compartments. **d** Composition of the TE library built de novo from the Bd21 genome (1,995 sequences) and proportions of TE insertion orders across pangenomic compartments

We used panREPET to identify shared TE insertions within *B. distachyon* genomes and compare the results with the literature. Figure 2a, b show the count of TE insertions shared across our reliable set of 42 accessions. Based on this distribution, we considered five pangenomic compartments: 1. Singleton TE insertions; 2. Cloud TE insertions shared by 2–7 accessions (2–20%); 3. Shell TE insertions shared by 8–38 accessions (20–95%); 4. Soft-core TE insertions shared by 39–41 accessions (95–98% of accessions) and 5. Core TE insertions shared by all accessions. A singleton TE insertion may reflect a recent individual-specific insertion, horizontal transfer, drift, or biased sampling. A cloud, shell, or soft-core TE insertion may correspond to an insert inherited from a common ancestor in a subset of related individuals. Core TE insertions are fixed insertions at the species level. panREPET identified 319,969 TE copies that cover their consensus by more than 95% (*covcons* = 95–105%). Among them, it detected 18,513 shared TE insertions including 595 core (1.2%), 1,139 soft-core (2.4%), 9,125 shell (19.4%), and 7,654 cloud (16.2%) while 28,497 are singleton TE insertions (60.6%). The vast majority of TE insertions appear to be singletons (Fig. 2a, b). The pipeline ran for 20 h on a 64-core 128-GB RAM virtual machine. Including partial copies (*covcons* = 75–125%), doubled the number of extracted copies to 653,695, corresponding to 35,922 shared TE insertions and 82,347 singletons. This resulted in a slightly higher proportion of core (1.4%) and singleton (69.6%) insertions (Fig. 2b). Longer TE copies tend to be singletons or occur in a few individuals (Fig. 2c). The pipeline ran for 3.6 days on a 64-core 128-GB RAM virtual machine.

**Fig. 3:**
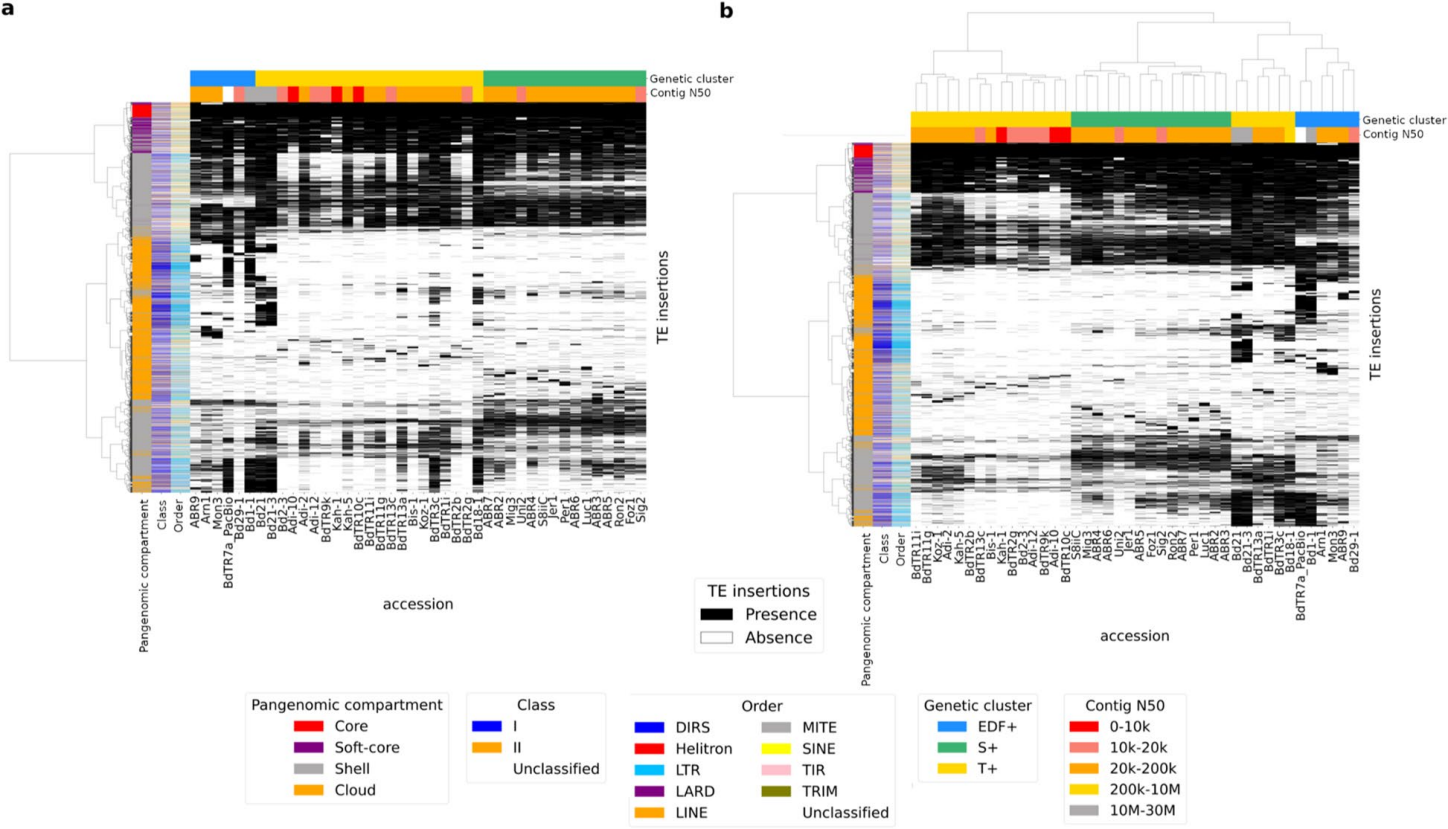
Clustermaps showing TE insertion presence/absence for each accession.**a** Accessions are vertically ordered according to a whole-genome SNP-based genetic tree from Gordon et al. [12], Supplementary Fig. 4a. **b** Accessions are vertically ordered according to their shared TE insertion patterns, after presence/absence-based clustering. The metric used is the Euclidean distance with Ward’s algorithm

We built a clustermap showing the TE copy presence or absence for each accession (Fig. 3a, b). Gordon et al*.* identified three genetic clusters based on > 3 million SNPs [12]: (i) EDF + with an extremely delayed flowering phenotype; (ii) T + from the Eastern Mediterranean, predominantly Turkish; and (iii) S + from the Western Mediterranean, predominantly Spanish. As a validation, we observed that relationships between accessions based on whole-genome SNPs are similar to those inferred from shared TE insertions (Fig. 3a, b). However, the TE insertion-based tree (Fig. 3b) fits even better with the gene presence/absence clustermap from the Gordon et al*.* study [12] (tree not shown); indeed, we observed the same two distinct sub-clusters in the cluster T +. We also examined the number of singleton TE insertions in relation to assembly quality metrics (Additional file 1: Fig. S1, Additional file 1: Fig. S2). The distribution of singleton insertions is not uniform among accessions and does not appear to follow genetic clusters (Additional file 1: Fig. S1). The number of singleton insertions decreases with assembly quality (Additional file 1: Fig. S2). Among the assembly quality metrics, contig N50 best explains this pattern (linear regression: contig N50 as regressor, singleton TE insertions as response variable; R^2^ = 0.191, *p* = 1.190e − 03). Accessions with better assemblies contain fewer singleton TE copies on average (Additional file 1: Fig. S1), likely because their copies are better detected and therefore easier to assign to cliques.

Singleton, cloud, and shell TE insertions mostly belong to LTR families, and LTR families represent a smaller fraction of insertions present in more individuals (Fig. 2d). Stritt et al*.* showed that the western ancestral population represented by the S + cluster lost retrotransposons as a result of several bottlenecks [28]. Supporting this hypothesis, the S + cluster shows low intra-diversity compared to the other genetic clusters (Fig. 3a). For soft-core and core TE insertions, the proportions of MITEs, LINEs, and SINEs increase as insertions are shared among more accessions (Fig. 2d). A study in wheat has already shown high sequence conservation of MITEs [29]. Horvath et al*.* showed, for 320 *B. distachyon* accessions, that retrotransposons are strongly correlated with demographic structure, whereas DNA transposons are weakly correlated [15]. This corroborates our observations and supports the validity of the panREPET method: core and soft-core TE insertions are mainly composed of DNA transposons, particularly MITE families, whereas shell, cloud, and singleton insertions are mostly composed of LTR families and thereby shape intra-specific diversity (Fig. 2d).

### Benchmarking panREPET

**Fig. 4:**
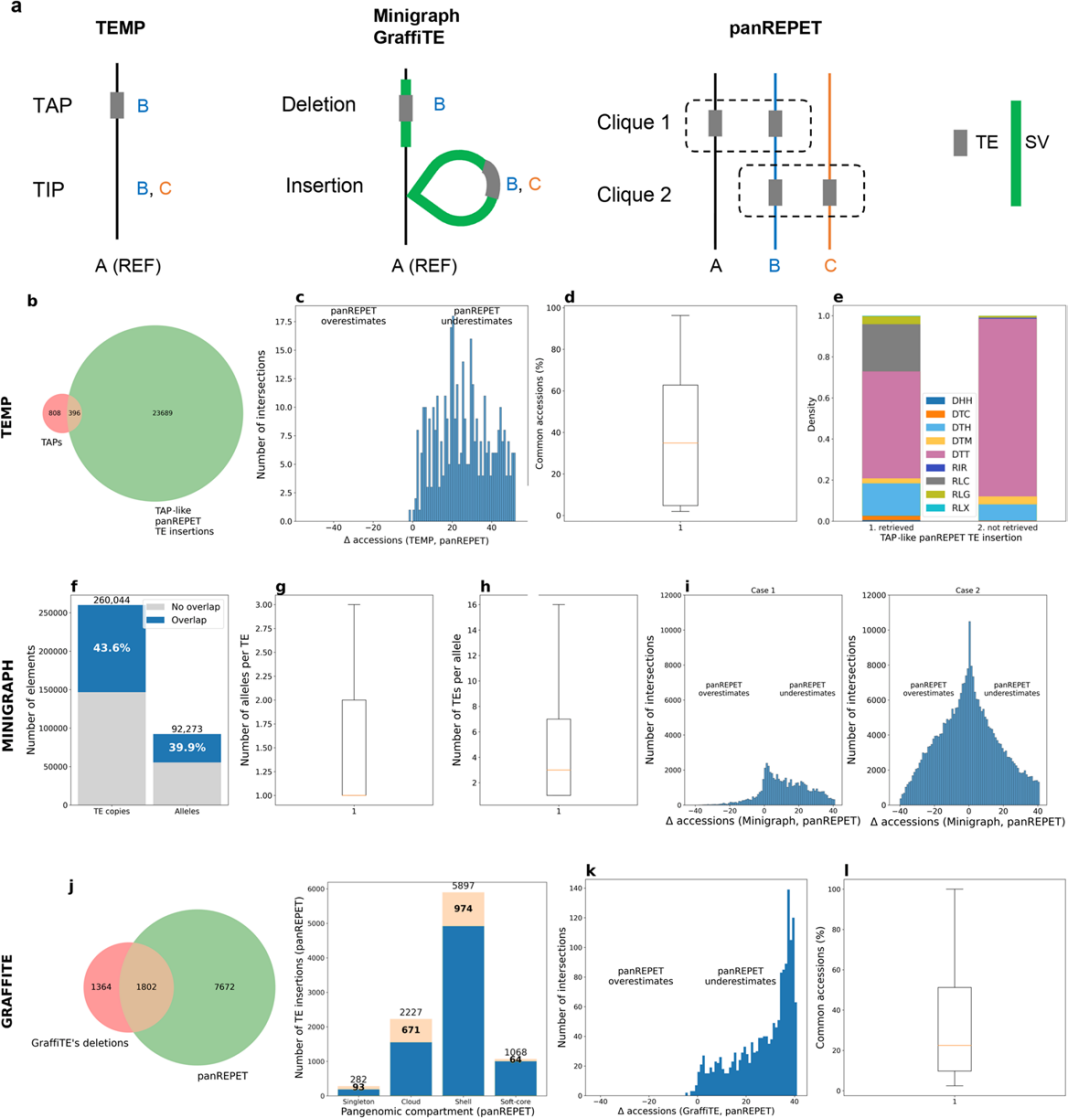
panREPET’s benchmark results. **a** Schematic representations showing the output of the alternative reference-centered tool and how it relates to panREPET, which is reference-free. A, B, and C represent genomes. TEs are shown in grey and SVs in green. A clique corresponds to a shared TE insertion among genomes. **b** Venn diagram showing TAPs identified by TEMP that intersect with TAP-like panREPET TE insertions. **c** Histogram of differences (Δ) in the number of accessions detected by TEMP minus those detected by panREPET for each common TE insertion. **d** Proportion of common accessions among TE insertions detected by both TEMP and panREPET. **e** Comparison of TE orders for TAP-like panREPET TE insertions retrieved by TEMP versus those not retrieved.** f** Bar plots showing the proportion of panREPET TE copies that intersect alleles from Minigraph and vice versa. **g** Boxplot showing the distribution of the number of alleles per TE copy. **h** Boxplot showing the distribution of the number of TE copies per allele.** i** Histograms of differences (Δ) in the number of accessions sharing the Minigraph allele minus those sharing the corresponding TE copy from panREPET. The allele is included within the TE copy (case 1). The TE copy is included within the allele (case 2). **j** Venn diagram (left) showing GraffiTE deletions that intersect deletion-like panREPET TE insertions. Bar plots (right) showing the proportion of panREPET TE insertions intersecting a GraffiTE deletion in each pangenomic compartment. **k** Histogram of differences (Δ) in the number of accessions detected by GraffiTE minus those detected by panREPET for each common TE insertion. **l** Boxplot of the distribution of the proportion of common accessions at GraffiTE and panREPET copy intersections. REF: reference; SV: structural variant; TE: transposable element; TIP: TE insertion polymorphism; TAP: TE absence polymorphism

We compared panREPET with several alternative approaches used in recent TE pangenomic studies. We first compared our approach to TEMP, which relies on paired-end read mapping [30], because it was used by Stritt et al*.* [28] to analyse TE polymorphisms in the same dataset used in our study. Additionally, we compared panREPET with two other approaches that detect structural variants (SVs) in whole-genome assemblies: Minigraph [31] and GraffiTE [32], to assess whether identifying TE copies through SV detection is limiting, given that Minigraph is not TE‑centric and GraffiTE was specifically developed for this purpose. We excluded core TE copies from panREPET, as our aim was to compare TE copies shared by subsets of accessions. ​​The three tools reported coordinates relative to the reference genome, whereas panREPET reported coordinates independently for each genome (Fig. 4a). Further details are provided in Additional file 6: Supplementary Results, detailed benchmarks of panREPET.

TEMP tool reports a “TE absence polymorphism” (TAP) as a TE position in the reference assembly that is missing in at least one other accession (Fig. 4a). In panREPET, it relates either to a TE insertion shared with the reference but absent in at least one other genome, or to a singleton TE copy present in the reference. Hereafter, we refer to this as a “TAP-like panREPET TE insertion”. TEMP detects only 1.6% (396 out of 24,085) of the shared TE insertions identified by panREPET, whereas panREPET recovers 32% (396 out of 1,204) of those detected by TEMP (Fig. 4b). Overall, panREPET identifies a larger number of shared TE insertions (Fig. 4b). TEMP overestimates the number of accessions sharing a given TE insertion, whereas panREPET underestimates it. On average, TEMP reports 25 more accessions per shared TE insertion than panREPET, with a median difference of 25 accessions as well (Fig. 4c). On average, 39.4% of accessions are shared between pairs of TE insertions detected as the same by both tools, and the median proportion is 38.2% (Fig. 4d). By comparing TAP-like panREPET TE insertions that are retrieved by TEMP with those that are not (Fig. 4e), we observe that Copia elements (RLC) are predominantly recovered, whereas TIR elements (DTT) are largely missing. The differential recovery of Copia (RLC) and TIR (DTT) elements by TEMP likely reflects its bias toward detecting larger LTR retrotransposons, whereas shorter DNA transposons are less reliably captured by paired-end mapping. TEMP also reports a “TE insertion polymorphism” (TIP) as a TE not shared with the reference genome (Fig. 4a). This comparison is not possible because TIP positions in accessions other than the reference are unknown.

The Minigraph tool reports SVs and identifies the accessions that share each variant (Fig. 4a). The main question we aim to address is how fragmented the panREPET-identified copy is within these SVs. Of the 260,044 TE copies detected by panREPET across the 42 accessions (*covcons* = 95–105%), 43.6% overlap a Minigraph allele with at least 10% coverage between the allele and the TE copy (Fig. 4f). To evaluate the level of fragmentation in regions where Minigraph alleles intersect panREPET TE copies, we quantified the average number of alleles intersecting a TE copy (Fig. 4g) and the average number of TE copies intersecting an allele (Fig. 4h). Because alleles are on average larger than TE copies (Additional file 1: Fig. S3a-b), it is expected that the average number of TE copies per allele would exceed 1, and we indeed find a mean of 7 and a median of 3 (Fig. 4h). This corresponds to large SVs containing several TE copies. We also observe an average of 1.1 alleles per TE copy (Fig. 4g), indicating that some TE copies are fragmented into multiple alleles. We defined case 1 as intersections covering more than 100% of the allele, and case 2 as intersections covering more than 100% of the TE copy. In case 1, but not in case 2, panREPET tends to underestimate and Minigraph to overestimate the number of accessions (Fig. 4i). Altogether, these observations indicate that when the TE copy is incompletely detected, Minigraph loses specificity.

The GraffiTE tool reports a deletion as a TE copy that is absent in an alternative genome but present in the reference. While GraffiTE identifies TEs in alternative assemblies through SVs, panREPET directly detects them on whole-genome assemblies (Fig. 4a). We find that 54% (1,802/3,166) of deletions detected by GraffiTE are retrieved by panREPET, whereas only 19% (1,802/9,474) of panREPET copies are retrieved by GraffiTE (Fig. 4j, left). GraffiTE also detects singleton and cloud TE copies (32% and 30%, respectively) more readily than shell and soft-core TE insertions from panREPET (16% and 5.9%, respectively) (Fig. 4j, right). GraffiTE overestimates the number of accessions compared to panREPET, with a mean difference of 26 and a median of 31 (Fig. 4k). On average, 33% of accessions are shared between pairs of TE insertions detected as the same by the two tools, with a median proportion of 24% (Fig. 4l). GraffiTE also reports an insertion as a TE copy present in at least one alternative genome but absent in the reference. We cannot compare insertions because genomic coordinates for alternative genomes are not directly reported by GraffiTE.

The three alternative tools chosen for the benchmark tend to overestimate the number of accessions sharing a TE insertion, while panREPET appears more specific (Fig. 4c, i, k). Our bidirectional detection approach is likely more stringent, allowing the elimination of false positives and thereby allowing more accurate detection of real cases.

### Core TE insertions

**Fig. 5:**
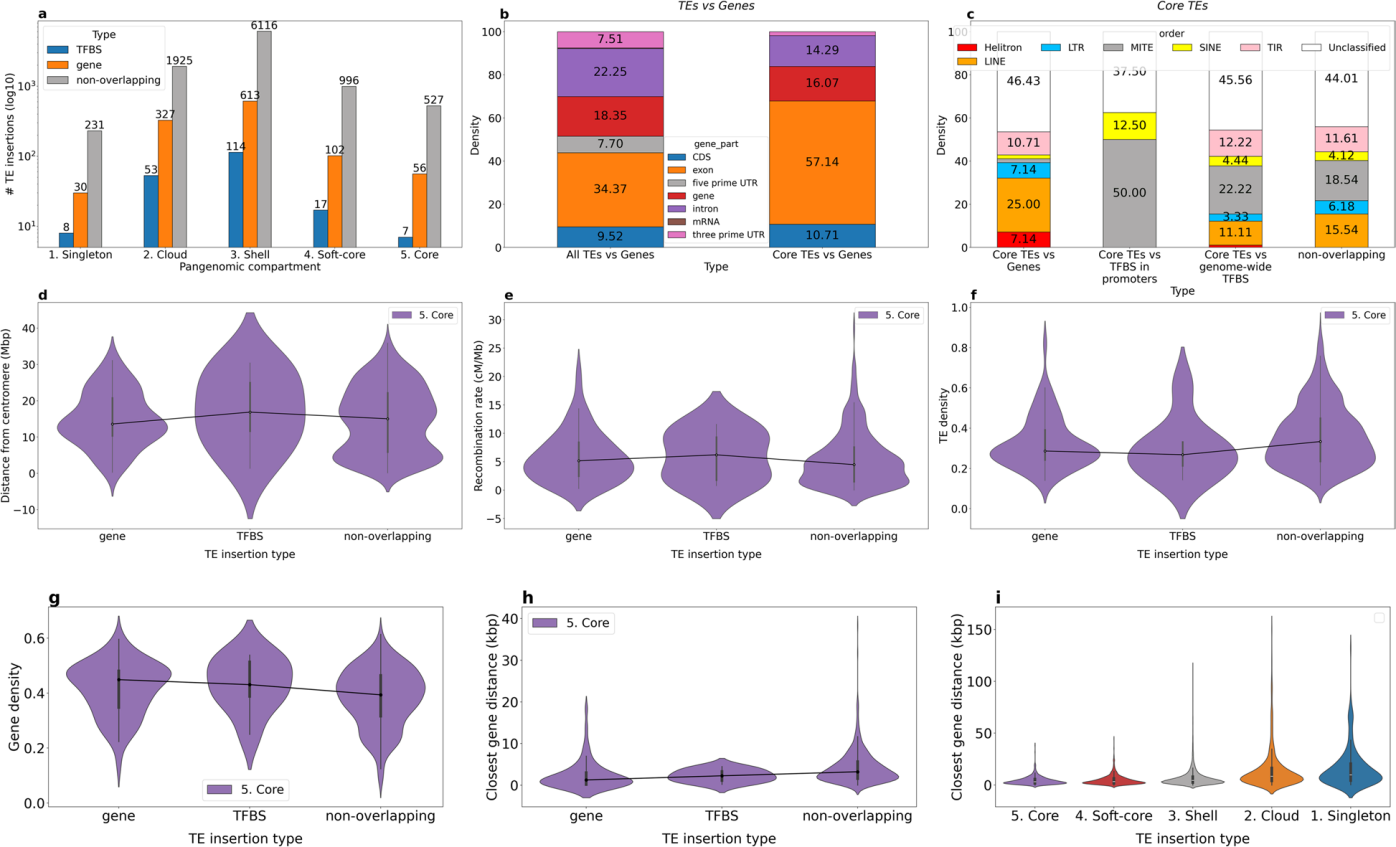
Analysis of core TE insertions on the Bd21 genome. **a** Log₁₀-transformed number of TE insertions intersecting genes or TFBSs, grouped by pangenomic compartment. **b** Stacked bar plots showing the proportions of TE insertions from the Bd21 genome intersecting gene features by at least 80%. Genome-wide TE insertions and core TE insertions are compared. Only the gene feature with the greatest overlap is considered. **c** Stacked bar plots showing the proportions of TE orders among core TE insertions that overlap genes, promoter-region TFBSs, genome-wide TFBSs, or do not overlap genes or TFBSs. **d-h** Distributions of the following parameters for core TE insertions overlapping genes, overlapping promoter-region TFBSs, or not overlapping genes or TFBSs: **d** distance to the centromere (Mbp), **e** recombination rate (cM/Mb),** f** TE density, **g** gene density,** h** distance to the nearest gene (kbp). **i** Distance to the nearest gene (kbp) for TE insertions that do not overlap genes or TFBSs across all pangenomic compartments. TFBS: transcription factor binding site

On the Bd21 genome, about 10% (56/589) of core TE insertions intersect a gene feature over at least 80% of their length (Fig. 5a). They mainly intersect exons (Fig. 5b). This supports the idea that ancient TEs may have been co-opted by host genomes, contributing to novel exon formation [33–35].

Across all pangenomic TE compartments, between 1.3% and 4% of transcription factor binding sites (TFBSs) in promoter regions (− 500 bp to + 100 bp relative to the transcription start site, TSS) from the PlantRegMap database [36] intersect a TE (Fig. 5a). To test for genome-wide significance, we shuffled the 17,589 predicted promoter TFBSs on the Bd21 genome using *bedtools shuffle* [24] and counted how many overlapped core TE insertions with 100% coverage on the TFBS. Under the null hypothesis, any overlap between core TE insertions and shuffled TFBSs occurs purely by chance, consistent with a random distribution based on their genomic abundances. Repeating this procedure 100 times showed that shuffled TFBSs do not intersect the core TE insertions by chance (chi-square goodness-of-fit = 722, *p* = 2.05e − 95, degrees of freedom = 99). We conclude that there is a statistically significant association between core TE insertions and promoter-region TFBSs, suggesting that some core TEs result from the co-optation of TE-encoded TFBSs [23]. Concerning the 144,761 genome-wide predicted TFBSs (i.e., all TFBSs including those not in known promoter regions), we observed a statistically significant association between core TE insertions and all TFBSs (chi-square goodness-of-fit = 8,028, *p* < 1e-300, degrees of freedom = 99). In the following, we focus only on promoter-region TFBSs. We observed that core TEs harboring TFBSs are mostly MITEs (Fig. 5c), consistent with findings in maize, where domesticated MITEs carrying TFBSs appear to be involved in husk tissue-specificity [37].

For the remaining core TEs that do not overlap genes or TFBSs (527/589; Fig. 5a), we asked whether their conservation at the species level could reflect domestication through their role as essential structural components of centromeres [38, 39] and in maintaining centromeric and telomeric stability, as well as in heterochromatic silencing [40]. To test this, we mapped their chromosomal positions (Additional file 1: Fig. S4) and examined their distance to the centromere, the recombination rate at the insertion site (see Methods, *Brachypodium distachyon* data), the local TE and gene densities, and their distance to the nearest gene (Fig. 5d–h). We performed an ANOVA to compare these distributions between core TEs that do not overlap genes or TFBSs and the other core TE insertions. Core TEs that do not overlap genes or TFBSs are significantly closer to the centromere than those that do (Fig. 5d; ANOVA, *p* = 3.648e − 03). TE insertions are also more likely to settle in non-recombining regions through fixation [41], as they may have been fixed by genetic drift [42, 43]. Core TEs that do not overlap genes or TFBSs are not more frequent in low-recombination regions than the other core TEs (Fig. 5e; ANOVA, *p* = 0.4576). Heterochromatic regions are also less subject to recombination [44], and TEs tend to accumulate there [45–47]. To infer the chromatin state of these genomic regions, we computed TE and gene density per 1-Mbp interval across chromosomes (Fig. 5f, g). Core TEs that do not overlap genes or TFBSs are not preferentially located in heterochromatin regions (Fig. 5f, g; ANOVA,* p* = 0.1395 for TE density and* p* = 0.0516 for gene density) and lie farther from the nearest gene than other core TEs (Fig. 5h; ANOVA, *p* = 4.751e − 03).

As shown in Fig. 5i, singleton and cloud TE insertions that do not overlap genes or TFBSs lie farther from the nearest gene than their cognate core and soft-core insertions, which likewise do not overlap genes or TFBSs (t-test: t = − 19.96, *p* = 2.54e − 84). This suggests that core TEs without gene or TFBS overlap may be conserved through the effect of a nearby gene under positive selection [46, 48].

### TE insertion dates

The age of TE insertion events can reveal their evolutionary dynamics over time. By combining pairwise genetic distances among accessions with our reference-free pangenome-wide TE analysis, we were able to obtain rough TE-insertion age estimates that better reflect divergence among accessions than existing methods. In addition, we consider all types of TE copies, not only LTR elements, unlike previous methods [49–52]. Some other methods date TE copies using percent identity to the consensus sequence as a proxy for age: the more a copy diverges from its consensus, the older it is assumed to be [53, 54]. For all TE copies detected across accessions, we plotted the distribution of percent identity to the consensus sequence, grouped by the number of accessions sharing each TE insertion (Additional file 1: Fig. S5). As expected, core TE insertions show low identities to their consensus (< 65%), consistent with ancient and conserved copies.

**Fig. 6:**
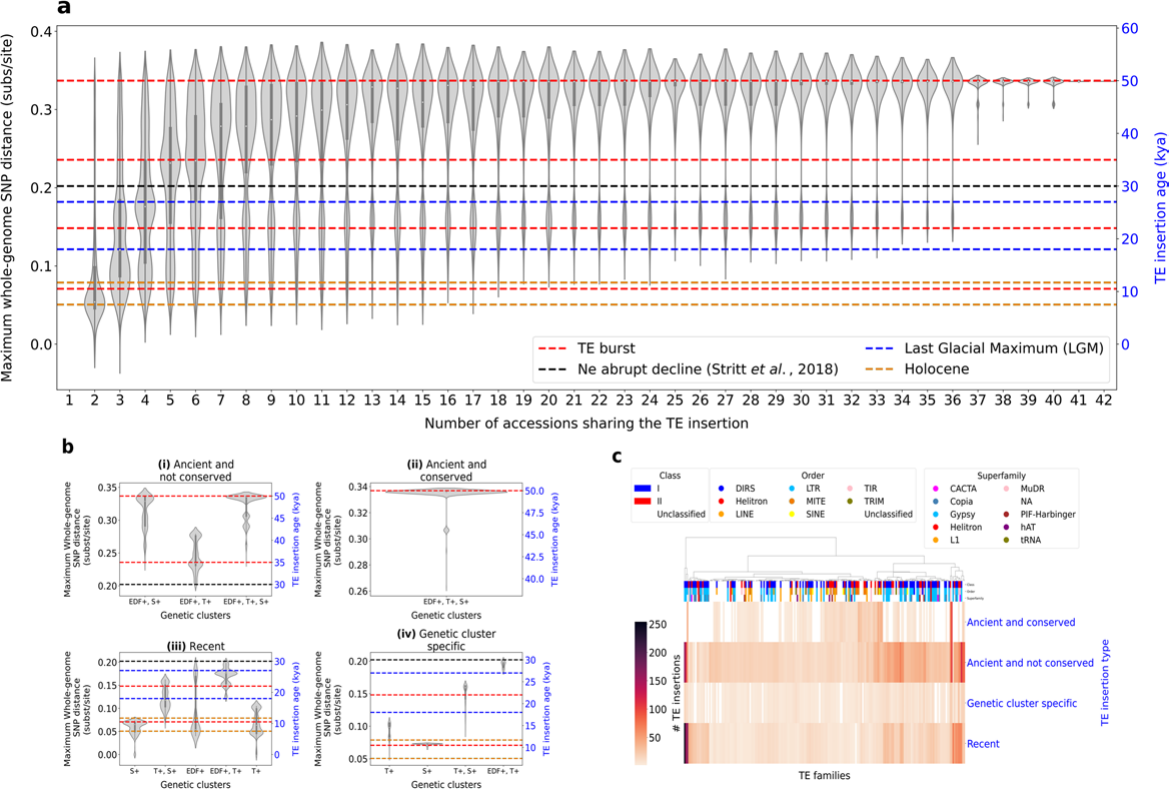
**a** Boxplots showing the age distribution of TE insertions grouped by the number of accessions sharing them. Age is estimated from the maximum pairwise whole-genome SNP distance between accessions in a given TE clique. Red dashed lines mark the regions where the violin plots are widest, corresponding to peaks in TE insertion density. The widest sections of the violin plots are zoomed in Fig. 6b. **b** Boxplots showing the age distribution of TE insertions according to the genetic clusters to which the accessions sharing each insertion belong. This panel corresponds to Fig. 6a split into four TE insertion categories: (i) ancient but not conserved insertions; (ii) ancient and conserved insertions; (iii) recent insertions; and (iv) insertions that arose after cluster divergence. **c** Clustermap showing TE family abundance according to TE insertion categories. Euclidean distance with Ward’s algorithm was used for clustering. Only families with more than 20 copies were retained for readability

However, this approach is limited because it relies on consensus sequences from the reference TE library. We therefore introduce a SNP-based method to estimate TE insertion age that accounts for species-wide evolutionary divergence. TE insertions shared among accessions must predate lineage splits, and their age can be approximated by the maximum SNP-based genetic distance between any two accessions sharing the insertion, which provides a lower-bound divergence time. We observe that the more an insertion is shared, the greater its SNP-based genetic distance (Fig. 6a). The plateau corresponds to the maximum SNP distance among accessions (0.335 substitutions/site), which reflects the distance between the two most divergent accessions, Arn1 and Ron2 (see Gordon et al*.* [12], Supplementary Fig. 4a [12]). Minadakis et al*.* estimated genetic cluster divergence using a multispecies coalescent approach [13]: the S +, T +, and EDF + clusters diverged at least 45 kya, and the S + and T + clusters split more recently, around 23 kya. To convert substitutions per site into kya, we assumed that the maximum whole-genome SNP distance between S + and T + accessions (0.1549 substitutions/site, observed between Bd21 and Sig2) corresponds to 23 kya.

Figure 6a illustrates four types of TE insertion events among the 18,513 shared TE insertions. (i) *Ancient but not conserved insertions* are shared by a few to many accessions (2–36 accessions), with at least two accessions being genetically distant on the whole-genome SNP tree (> 0.20 substitutions/site, ~ 30 kya). We identified 9,824 such cases. Note that the accessions sharing the largest number of these insertions (Bd1-1, Bd18-1, Bd21, Bd21-3, BdTR3c, BdTR7a) have higher-quality assemblies (Additional file 1: Fig. S1, Fig. 3), likely because the corresponding regions are better assembled. This category is abundant, and the prevalence of purifying selection reported for *B. distachyon* TEs [15] is consistent with this observation. (ii) *Ancient and conserved insertions* are shared by 37–42 accessions, suggesting that they predate speciation. We identified 2,296 such insertions. (iii) *Recent insertions* are shared by only 2–6 accessions, which cluster closely on the SNP tree (< 0.20 substitutions/site, ~ 30 kya). We identified 4,890 such cases. Most of these are shared by Arn1 and Mon3 from Spain, which share a mixed ancestry [15], consistent with the large number of specific insertions they share. (iv) *Insertions that arose after the divergence of intra-specific genetic clusters* are restricted to accessions within the same clade. We identified 1,503 such insertions.

We dated four major transposition events in *B. distachyon*. A first major transposition event, at least 45 kya, may correspond to TE insertions that occurred before speciation or during the divergence of the three lineages. A second major transposition event around 35 kya involves TEs mostly shared by EDF + and T + (Fig. 6b). This pattern suggests that the S + genetic cluster experienced a bottleneck at that time, with the few surviving S + individuals potentially losing their copies through genetic drift or selection. A third major transposition event occurred at 22 kya, during the Last Glacial Maximum (LGM) [55], a period associated with thermal stress. As shown in Fig. 6a, b, TE insertions show an apparent gap around 30 kya, which may reflect the strong demographic bottleneck reported for that period [13, 28]. This bottleneck was followed by a population expansion in the recent past [13, 28], potentially creating permissive conditions for renewed TE activity around 22 kya. In this context, reduced effective population size following a founder event can diminish the efficacy of purifying selection, facilitating TE accumulation and activation [56, 57]. Finally, around 10 kya, a burst of transposition appears to have occurred during the Holocene (11.7–7.5 kya), a period marked by deglaciation in Europe and associated species range shifts [13]. Among recent events, we also detect a burst in S + at 11.7 kya (Fig. 6b). S + may have experienced additional transposition bursts during its recolonization of Europe and the Middle East.

Identifying high-copy active TE families helps clarify their impact on genome evolution [58]. To pinpoint the families contributing to the four major transposition waves, we examined TE family abundance by insertion type (Fig. 6c). The oldest conserved insertions, dating to at least 45 kya, are dominated by MITE, LINE, TIR, and SINE families. LTR families, by contrast, are mostly either recent or ancient but not conserved, pointing to strong turnover; the two left-most LTR families in Fig. 6c correspond to Ty3/Gypsy. Stritt et al*.* similarly reported recent activity in centromeric Ty3/Gypsy and Ty1/Copia elements [52].

### Factors affecting the transposition dynamics of TE families

**Fig. 7:**
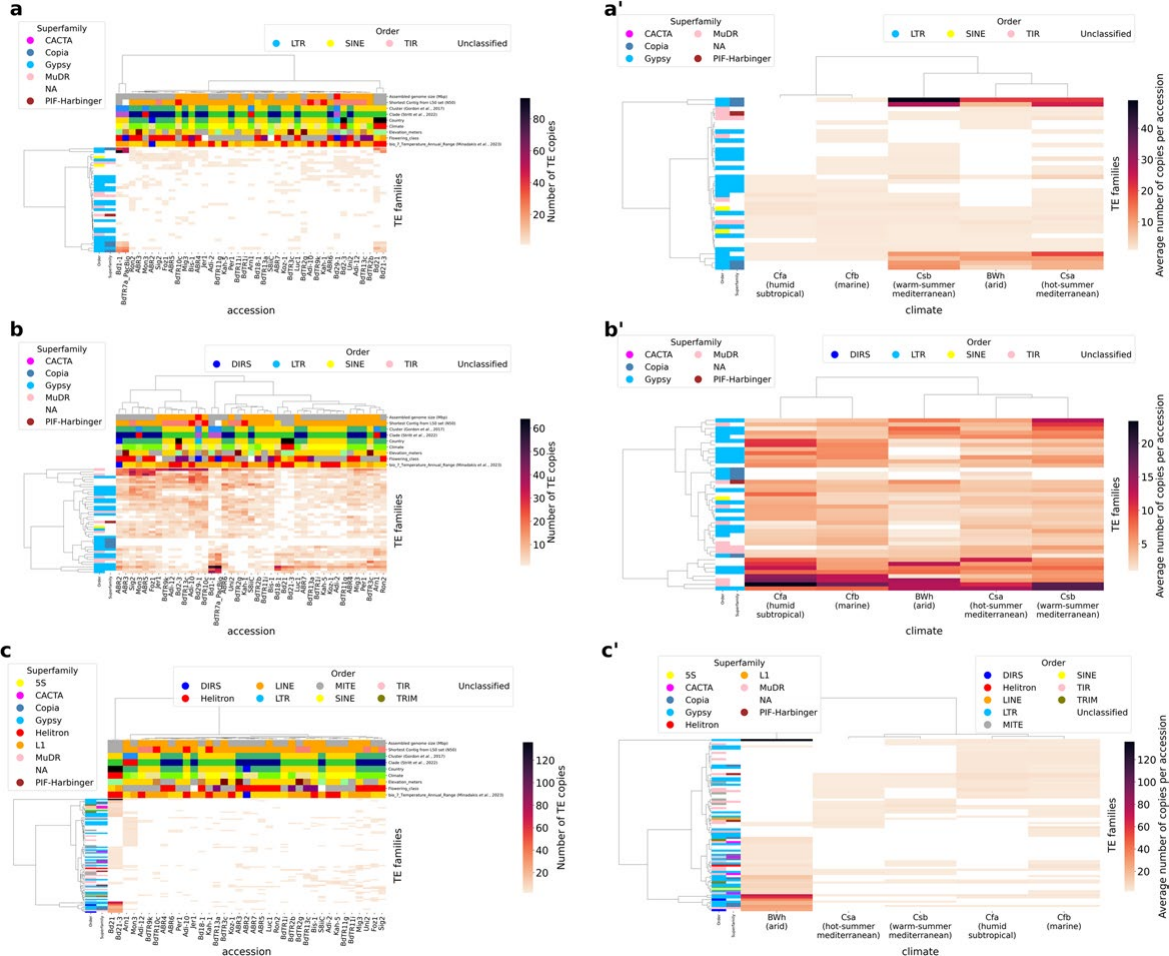
**a**, **b**, **c** Clustermaps showing the abundance of recent TE insertions per TE family and per accession. Accessions (x-axis) and TE families (y-axis) are clustered based on similarities in their TE family dynamics. Column color labels are detailed in Additional file 3: Table S2. Clustering was performed using Euclidean distance and Ward’s linkage. The percent coverage between each TE copy and its consensus is 95–105%. **a, b** Singleton TE copies. Only TE families with at least 10 insertions in at least one accession were retained.** a** TE copies with > 95% identity to their consensus. **b** TE copies with < 95% identity to their consensus. **c** TE copies inferred to have transposed after the Holocene (age < 7.5 kya). **a**’,** b**’,** c**’ Each clustermap corresponds to its counterpart; the only differences are that accessions are arranged by climate class on the x-axis and the values represent the average number of TE copies per accession to account for the unequal distribution of accessions across climate classes (Arid BWh: 3; Humid subtropical Cfa: 3; Marine Cfb: 14; Hot-summer Mediterranean Csa: 13; Warm-summer Mediterranean Csb: 9)

We investigated geo-climatic factors that may underlie TE mobilization, focusing on local conditions that could explain recent TE activity. To compare recent TE family dynamics among accessions, we clustered them by the number of recent TE family copies (Fig. 7). As a proxy, we defined recent TE insertions as singletons with > 95% identity to their consensus, a cutoff already used to identify young subfamilies [59] (Fig. 7a, a’), and other singletons (Fig. 7b, b’). A second proxy is TE insertions dated to after the Holocene (age < 7.5 kya; Fig. 7c, c’). We chose this threshold because *B. distachyon* moved southward during the last glacial period and later recolonized Europe and the Middle East within the last five thousand years (niche-modeling analysis in [13]).

Similar patterns of TE family dynamics across accessions may suggest shared determinants of TE activity (e.g., environmental stress). To identify such determinants, we examined for each accession its genetic cluster, clade, country, climatic classification, elevation, and flowering class. We also included annual mean precipitation, as this variable was reported to have the highest number of genes located in significantly associated regions [13]. Singleton TE family dynamics do not appear to be explained by genetic clusters but rather by other factors (Fig. 7a, b). Accessions with the highest contig-level N50 values cluster together (the pairs Bd1-1/BdTR7a from Turkey and Bd21/Bd21-3 from Iraq) (Fig. 7a). They contain fewer singleton TE insertions than the other accessions (Additional file 1: Fig. S1), yet the same families cluster together, which is not the case in the other accessions. The pairs Bd21/Bd21-3 and Bd1-1/BdTR7a specifically share Copia and Ty3/Gypsy LTR superfamilies as well as MuDR TIR superfamilies (Fig. 7a). This may suggest that accessions with low assembly quality show fewer copies, partly due to poorer annotation.

We also examined TE family dynamics across climate classes for TE insertions that occurred after the Holocene (age < 7.5 kya) (Fig. 7c’) to highlight transposition events potentially induced by abiotic conditions. To avoid biases due to the uneven number of accessions per climate class (Arid Bwh: 3; Humid subtropical Cfa: 3; Marine Cfb: 14; Hot-summer Mediterranean Csa: 13; Warm-summer Mediterranean Csb: 9), we plotted the average number of TE copies per accession. We observed that accessions from arid climates exhibit a clear over-representation of singleton copies, suggesting activation of specific TE families. Accessions from hot-summer and warm-summer Mediterranean climates also appear more similar to each other than to those from humid subtropical or marine climates (Fig. 7c’).

We then examined whether TEs potentially associated with local activation contained TFBSs. We intersected the 144,761 predicted genome-wide TFBSs with the supernumerary TE copies (TE families with more than 10 or 2 copies; Additional file 5: Table S4) and retained only TEs whose sequence overlapped a TFBS by 100% (Additional file 5: Table S4). We restricted this analysis to Bd21 because TFBS annotations were only available for this accession. Among the supernumerary recent TE insertions overlapping a TFBS, most belong to LTR families (Additional file 5: Table S4). We observed TFBSs targeted by OsMADS56, OsMADS4, OsMADS8 and OsMADS3, members of the MIKCc-type MADS-box gene family. Studies in *Oryza sativa* have shown that these genes are critical for reproductive organ development [60]. OsMADS8, in particular, is required for tapetum formation [61], and degradation of its protein by certain ambient air pollutants can lead to pollen sterility in petunia [62]. In addition, MYB-like DNA-binding domains, such as that of AtMYB002, are involved in drought responses in *Arabidopsis thaliana* [63].

## Discussion

### panREPET, a new method to improve TE analysis

Several pipelines exist for detecting TEs in pangenomes. They generally analyse TEs relative to a reference genome. Two approaches have been used to identify neo-TE insertions in resequenced genomes: (i) paired-end mapping (PEM), which detects discordant read pairs [64, 65], and (ii) split-read methods, which detect truncated reads corresponding to the junction between the TE insertion and the reference sequence. Unlike discordant reads, truncated reads can pinpoint the TE insertion at single-base resolution. However, split-read coverage is often low, and the method is highly sensitive to read length [10, 66]. In short, it cannot distinguish between intact and truncated copies across accessions at a given locus. The TEMP pipeline [28, 30] combines PEM and split-read evidence. When comparing panREPET with TEMP (Results, Benchmarking panREPET), we observed that panREPET detects more numerous shared TE insertions overall (Fig. 4b) and more effectively identifies DNA transposon polymorphisms (Fig. 4e). Quadrana et al*.* complemented the split-read approach with a validation based on the target site duplication (TSD) generated by most TE families upon insertion [9]. However, this method cannot be applied to TEs lacking TSDs, such as Helitrons. Overall, our results show that panREPET is able to analyse all major TE types accurately.

Short reads can identify the position of a TE insertion but do not provide access to its exact sequence composition. A TE copy is generally about 300 bp to 10 kbp long, which is often longer than the average short-read length of about 100–300 bp [67]. However, TE regulation analyses require knowledge of the exact regulatory sequences located on the TE sequence [68]. Some methods, such as the x-Transposable element analyzer (xTea), address the limitations of short reads by incorporating long-read data [69]. Another challenge is that aligning reads to a reference genome fails to capture the local genomic context of TE insertions in the genome from which the reads come. xTea overcomes this by using 10X Genomics’ Linked-Read technology, which barcodes DNA fragments originating from the same region to retain long-range context. Combined with long reads, this provides full TE reconstruction and richer information than short reads alone [69]. In contrast, our approach remains simple to apply and does not require any complex sequencing strategy, as it operates on multiple de novo assembled genomes and benefits from continuous improvements in sequencing and assembly technologies. panREPET provides direct access to the full TE sequence, its exact insertion length, and its precise genomic environment. For example, in our core TE insertion analysis, we were able to intersect TEs with genes and TFBSs and compute their exact coverage.

By comparing panREPET with Minigraph [31] (see Results, Benchmarking panREPET), we observed that approximately 40–50% of the TE copies detected by panREPET are retrieved by Minigraph (Fig. 4f, Additional file 4: Table S3). The remaining undetected TE copies may be explained by the fact that some TEs are not present within the detected SVs. We also observed that this kind of approach can lead to the detection of partial TE copies, and when a TE copy is only partially detected, Minigraph loses specificity. Indeed, because TE annotation occurs after SV discovery, the SV may truncate the TE copy at the outset (Additional file 1: Fig. S6). Annotating TEs first ensures that the complete sequence is detected. GraffiTE [32] uses multiple assemblies and/or long reads from the same species. It aligns each alternative sequence to the reference but does not perform pairwise alignments among the alternatives to detect SVs, and then annotates TE sequences within those SVs. We have chosen a different paradigm: we annotate TEs independently in each genome and then compare TE copies pairwise between accessions. Our approach ensures that only well-aligned and reliably annotated TE copies are compared across accessions, avoiding complications introduced by nested or structurally divergent insertions. The absence of a copy in one accession is also informative, as it reflects divergence or structural variation. When comparing panREPET with GraffiTE (see Results, Benchmarking panREPET), we observed that GraffiTE detects singleton and cloud TE insertions better than shell and soft-core ones (Fig. 4j). Finally, Minigraph and GraffiTE are reference-centric methods to detect SVs, while panREPET is designed to detect variations of TE insertions without relying on a reference (Fig. 4a). panREPET extracts copies from each assembly that may have undergone insertion or deletion events relative to their consensus at the TE copy scale. panREPET provides a detailed overview of how each nucleotide position has been preserved or altered since the TE insertion.

### Potential applications

When assemblies reach chromosome-level resolution, panREPET can distinguish translocation events from segmental duplications (paralogous copies, data not shown). In a translocation, a TE is present as a single copy in the genome but occupies different chromosomal locations across individuals; for example, a shared TE copy may lie on one chromosome in one individual and on a different chromosome in another. In contrast, a segmental duplication results in two copies of the TE within a genome. Our bidirectional detection approach identifies this situation when an individual carrying a shared TE insertion displays two best hits: one on the same chromosome and another on a different chromosome. Moreover, panREPET can process diploid genomes by taking the two phased haplotypes as input, hence highlighting haplotype-specific TE copies.

### The *Brachypodium distachyon* pangenome highlights transposable element dynamics in the species

A previous consensus library for *B. distachyon* was available from the TREP database and contained 233 sequences [28, 70], comprising 74% TIR, 20% LTR (10% Copia and 10% Gypsy), and 6% Helitron. Our consensus library, built de novo from the Bd21 reference (see Methods, TE library building), introduces additional TE families such as LINE, SINE, MITE and TRIM, thereby providing broader representation. Because MITEs and SINEs are the main components of the core and soft-core compartments (Fig. 2d), this library makes it possible to investigate ancient and conserved TE insertions that could not be studied previously. MITEs carrying TFBSs are conserved at the species level. For core TE insertions that do not overlap genes or TFBSs, our results suggest that their conservation may be driven by the effect of a nearby gene under positive selection.

In *B. distachyon*, panREPET detected a majority of singleton TE insertions (Fig. 2a, b). This is expected, as we extracted full (or nearly full) TE copies (see Results, panTEannot: a TEannot adaptation), and the number of singleton insertions may depend on assembly quality (Additional file 1: Fig. S2). Interestingly, Stritt et al*.* suggested that *B. distachyon* expands its populations without inter-accession breeding [12, 52], a process that may lead to an excess of singletons [6].

Our approach aims to understand how individual TE lineages have evolved at the species level. A TE insertion shared by only a subset of individuals can arise through two main scenarios. First, the insertion may be recent and therefore inherited only by closely related accessions. Second, the insertion may have been present in an ancestor but subsequently lost in some accessions through excision or ectopic recombination, with genetic drift or purifying selection fixing the loss in the population. To distinguish these possibilities, TE insertion age can be estimated. A molecular clock of 1.3 × 10⁻⁸ substitutions/site/year is typically used for LTR retrotransposons [49], but this does not account for the species’ evolutionary history and cannot be applied to all TE types. Stritt et al. estimated insertion ages in the same 54 *B. distachyon* genomes, but only for LTR copies [52]. In contrast, panREPET aims to date all TE types. With a whole-genome SNP tree [12] and coalescent-based cluster divergence times [13] available (see Results, TE insertion dates), we were able to date TE insertions using pairwise SNP distances, thereby accounting for genome-wide evolution and species-specific demographic events such as bottlenecks. However, we acknowledge that this approach can only propose a rough estimate of the true insertion age and may underestimate it, as the TE may have been inserted in an ancestor predating the divergence of the accessions.

We dated TE insertions before and after the Ice Age and identified four ancient bursts of transposition in *B. distachyon*. The first occurred at least 45 kya, before or during the split into the three lineages. panREPET requires assembled genomes as input, but the available Italian genomes do not meet this requirement. Including them would allow more precise dating since they represent the ancestral clade [13, 14]. A second burst occurred around 35 kya and may correspond to the bottleneck experienced by the S + genetic cluster. A third occurred around 22 kya during the Last Glacial Maximum, following the abrupt reduction in effective population size around 30 kya. The most recent major burst occurred around 10 kya during the Holocene, coinciding with the recolonization of Europe and the Middle East.

After the Holocene, the arid-climate accessions Bd21 and Bd21-3 appear to have activated specific TEs, potentially triggered by abiotic stresses such as pollution or drought (Fig. 7c’). With TFBS data available only for Bd21, our knowledge of copies not shared with this accession remains limited. A more even distribution of accessions across climate classes would also allow clearer inference of local activation patterns, although functional validation will ultimately be required to confirm the role of candidate TEs in adaptation. panREPET aims to facilitate this type of automated query for large datasets.

## Conclusion

We developed and present here a new bioinformatics tool, called panREPET, that makes it possible to retrace TE insertions in a species more accurately than existing approaches because: (i) it provides exact genomic coordinates of shared TE insertions for all individuals and their full sequences without relying on a reference genome, allowing the description of nucleotide preservation after insertion; (ii) it performs comparisons at the TE scale rather than the SV scale; and (iii) it facilitates dating all types of TE copies, whereas existing methods most date only LTR families. Finally, we dated two major TE bursts corresponding to key climate events and found specific TE families that seem to be activated in arid climates, and induced by pollution or drought after the Holocene.

## Methods

### Brachypodium distachyon data

We downloaded fifty-four de novo whole-genome assemblies of *B. distachyon*, along with their gene annotations and gene ontology information, from the Phytozome database (https://phytozome.jgi.doe.gov/) [12]. The BdTR7a genome is from *Stritt *et al*.* (2020) [52]. The genome versions are detailed in Additional file 2: Table S1.

We downloaded 17,589 predicted promoter-region TFBSs (− 500 bp to + 100 bp relative to the transcription start site) and 144,761 genome-wide predicted TFBSs from the PlantRegMap database, which uses the FunTFBS algorithm (https://plantregmap.gao-lab.org/download.php) [36]. Data are only available for the Bd21 reference. Gene ontology terms associated with these TFBSs were inferred using the annotation summary provided by Phytozome (Bdistachyon_556_v3.2.annotation_info.txt).

We fetched centromeric coordinates on Bd21 v3 from Li et al. [71], Table 1 [71]. Recombination rate data from Huo et al*.* [72] were remapped to the *B. distachyon* version 3 reference genome. For each SNP, we extracted its flanking sequence, including the polymorphic site written as [Bd21/Bd3-1] (e.g., [A/G]), and kept only the Bd21 allele. The resulting sequence was aligned to the v3 genome using global pairwise alignment (pairwise2.align.globalxx from Biopython v3.10.13) [73]. The new start and end positions were derived from the alignment and used to update SNP coordinates. Recombination rates (in cM/Mb) were calculated between consecutive SNPs located on the same chromosome, using their genetic distance (in cM) and physical distance (in Mb) based on their mapped v3 coordinates.

We extracted whole-genome SNP distances from the phylogenetic tree in Newick format from Gordon et al. [12], Supplementary Fig. 4a [12]. We converted the Newick format to pairwise distances between accessions using the *Phylo* module from Biopython v3.10.13 [73].

We used the kgc R package v1.0.0.2 [74] to associate each *B. distachyon* accession location with a general climate by converting the geographic coordinates into decimal degrees.

### TE library building

We built a de novo TE library from the reference Bd21 v3.2 with the TEdenovo pipeline [16] from REPET v3.0 (https://urgi.versailles.inrae.fr/Tools/REPET). The clustering step was done with GROUPER [19] only. The TE library is composed of consensus sequences which are reference sequences. We curated the annotation automatically by a second TEannot process [18] reducing the number of consensus sequences from 4,475 to 1,995. The TE classification comes from the classification of their consensus performed by the PASTEC classifier from the REPET package v3.0 [75]. The classification of some consensus sequences was inferred from other sequences within the same MCL (Markov Clustering) cluster (see REPET documentation, REPET V3.0 tutorial, 2025 [76]).

### panTEannot performance analysis

We annotated the fifty-four genomes of *B. distachyon* with the TEannot pipeline from REPET v3.0 without mreps [17].

We calculated sensitivity and specificity following Baud et al*.* [23]. True positives (TP) correspond to predicted TE nucleotides that truly belong to a TE copy, while false positives (FP) are predicted TE nucleotides that do not. True negatives (TN) are nucleotides correctly predicted as non-TE, and false negatives (FN) are true TE copy nucleotides missed by the TE prediction process. Sensitivity (true positive rate), given by the formula TP/(TP + FN), is obtained as the fraction of nucleotides in the predicted TE overlapping with the TE reference annotation. Specificity (true negative rate), defined as TN/(TN + FP), is more difficult to estimate for TEs because TN and FP require knowing all TE copies in the genome, which is unrealistic. As an approximation, we assume that gene coding sequences (CDS) are not TEs and not derived from TEs. Although TEs can occasionally overlap genes, this is expected to be rare, especially within CDS regions, which exclude introns and 5' and 3' UTRs where TEs are more frequent. Under this assumption, FP are predicted TE nucleotides overlapping a CDS, and TN are CDS nucleotides not predicted as TEs. Accuracy, defined as (TP + TN)/(TP + TN + FN + FP), measures the overall rate of correct predictions.

### Benchmarking panREPET

To enable a direct comparison with the TEMP results of Stritt et al. [28], we performed a panREPET analysis on the same set of fifty-four genomes. For this analysis, we used version 2.0 of the *B. distachyon* reference genome. The *B. distachyon* TE consensus sequences were obtained from the TREP database (http://botserv2.uzh.ch/kelldata/trep-db/index.html) [70], as reported by Stritt et al. The minimum coverage required between TE copies and their consensus was set to 80%.

For comparison with Minigraph and GraffiTE [31, 32], we used the panREPET results obtained for the 42 *B. distachyon* genomes (see Results, Retracing shared TE insertions). Minigraph depends on the order in which genomes are provided, as its graph construction proceeds incrementally. Therefore, we sorted the input genomes in descending order of assembly quality (using the assembled genome size after removing “N” occurrences as a compromise between contig N50 and scaffold N50; see Results, panTEannot). BdTR7a was used as the first genome because it is the only long-read assembly in the dataset. Minigraph was run with default settings, which include only structural variants affecting at least 50 bp. GraffiTE was also run with default parameters. We used the file “ALL.onecode.elem_sorted.bak” to retrieve the exact positions of TE sequences from alternative genomes based on the reference genome coordinates. For comparability with panREPET, we retained only the GraffiTE TE copies covering 95–105% of their consensus.

Since TEMP and GraffiTE do not directly provide genomic coordinates for alternative genomes, shared TE insertions can only be compared using their positions on the Bd21 reference genome. We intersected TAPs and deletions from TEMP and GraffiTE, respectively, on the Bd21 reference genome with the shared Bd21 TE copies identified by panREPET. A TE copy was considered retrieved when it was annotated with the same consensus sequence in both datasets. We used *bedtools intersect* from Bedtools v2.30.0 [24] to intersect the coordinates of TE insertions from TEMP, Minigraph, or GraffiTE with those from panREPET. To assess the proportion of individuals shared between the same TE insertion detected by two tools, we counted the individuals common to both insertions and divided this number by the larger of the two individual counts.

### Genomic analysis tools

We used *bedtools intersect* from Bedtools v2.30.0 to intersect TE, gene, and TFBS coordinates, configured with the –wo option to report the original A and B entries as well as the number of overlapping base pairs between the two features [24]. We generated clustermaps using the Seaborn package in Python v3.10.13 [77]. ANOVA analyses were performed with the statsmodels module in Python v3.10.13 [73]. T-tests were carried out using the SciPy library in Python v3.10.13 [78]. Dataframes were handled with the pandas library in Python v3.10.13 [79].

## Supplementary Information

Additional file 1: Figs. S1–S6.

Additional file 2: Table S1. Accession information and TE coverage. Genomes removed from the study are shown in bold and marked with an asterisk. The reference genome is indicated in bold.

Additional file 3: Table S2. Sheet 1: List of traits describing the 42 Brachypodium distachyon accessions. Color codes correspond to those used in Figure 7. Sheet 2: Climate traits for each accession, in raw format.

Additional file 4: Table S3. Intersection results between TE insertions detected by panREPET and alleles detected by Minigraph. TE copies covering 95–105% of their consensus are shown in black, and those with coverage between 75–125% are shown in blue.

Additional file 5: Table S4. Intersection results between genome-wide predicted TFBSs and recent, over-represented TE insertions.

Additional file 6. This file contains extended descriptions of the panTEannot and panREPET pipelines and benchmarking analyses.

## Data Availability

The Brachypodium distachyon (Bd21) TE library and TE annotation file (GFF3) are available in the RepetDB database (https://urgi.versailles.inrae.fr/repetdb/begin.do) [80] and have also been deposited in the recherche.data.gouv repository with the digital object identifier (DOI): 10.57745/L2TBDE [81]. All the analysis outputs have been deposited in the recherche.data.gouv repository with the DOI: 10.57745/L8XGV5 [82]. The source code for panTEannot, panREPET, and the analysis scripts is available at https://forge.inrae.fr/urgi-anagen/panTEannot [83], https://forge.inrae.fr/urgi-anagen/panREPET [84], and https://forge.inrae.fr/urgi-anagen/scripts_for_panrepet [85]; all are released under the CeCILL open source license. In addition, panTEannot, panREPET, and the accompanying analysis scripts are referenced in the open archive HAL (https://hal.inrae.fr/hal-05195049, https://hal.inrae.fr/hal-05195047, and https://hal.inrae.fr/hal-05199481, respectively) and are archived in the Software Heritage code repository (https://archive.softwareheritage.org/swh:1:dir:8a8d539918a9484fda4e17b9b3856c426be33525, https://archive.softwareheritage.org/swh:1:dir:c5869aafcd21acac329d6a0fb816938b55734e42, and https://archive.softwareheritage.org/swh:1:dir:4ece52a25918b63e4e008a2015143bad051a7222, respectively).
