# Supplementary material for: A reference-free pipeline for detecting shared transposable elements from pan-genomes to retrace their dynamics in a species
Source: Genome Biol. 2026 Feb 7;27:117. doi: 10.1186/s13059-026-03984-5 (PMC13059284; doi:10.1186/s13059-026-03984-5)
Supplement: Supplementary file 1 — Additional file 1: Figs. S1–S6. [file 13059_2026_3984_MOESM1_ESM.docx]

**Supplementary Information: Additional file 1**

**A reference-free pipeline for detecting shared transposable elements from pan-genomes to retrace their dynamics in a species**

Somia Saidi^1^, Mathieu Blaison^1^, María del Pilar Rodríguez-Ordóñez^1^, Johann Confais^1*^ & Hadi Quesneville^1^

^1^URGI - Université Paris-Saclay, INRAE, BioinfOmics, URGI, 78026, Versailles, France

*Corresponding author

Johann Confais: [johann.confais@inrae.fr](mailto:johann.confais@inrae.fr)

### Supplementary Figures


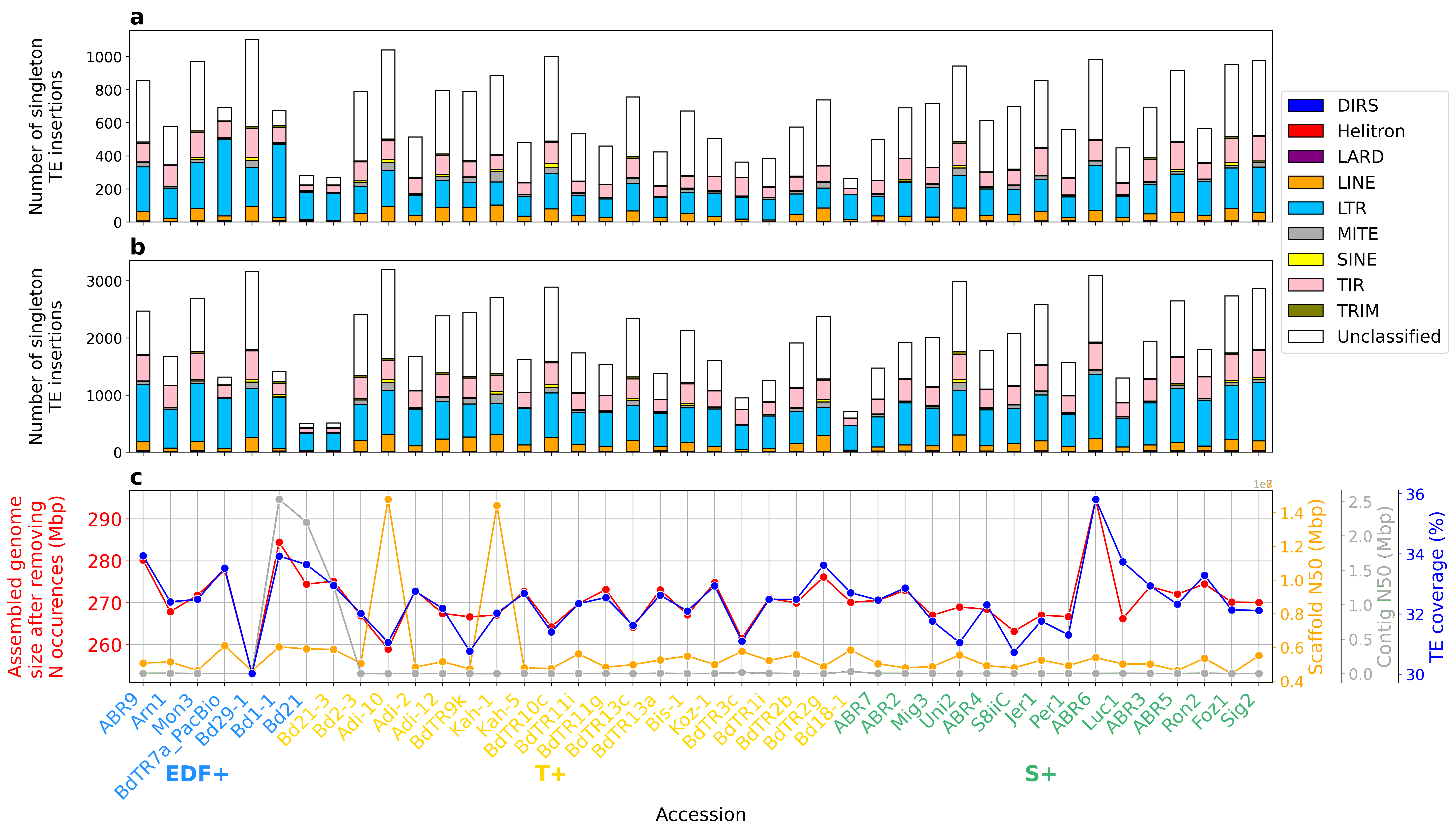


**Figure S1.** (a, b) Distribution of the number of singleton TE insertions across accessions. Accessions are ordered following the whole-genome SNP genetic tree from Gordon *et al.* (2017), Supplementary Figure 4a of that study. (a) The percentage coverage between the TE copy and its consensus (covcons parameter) is 95–105%. (b) covcons = 75–125%. (c) Assembled genome size after removing “N” bases (Mbp), scaffold N50 (Mbp), contig N50 (Mbp), and TE coverage from panTEannot (%) of each accession.


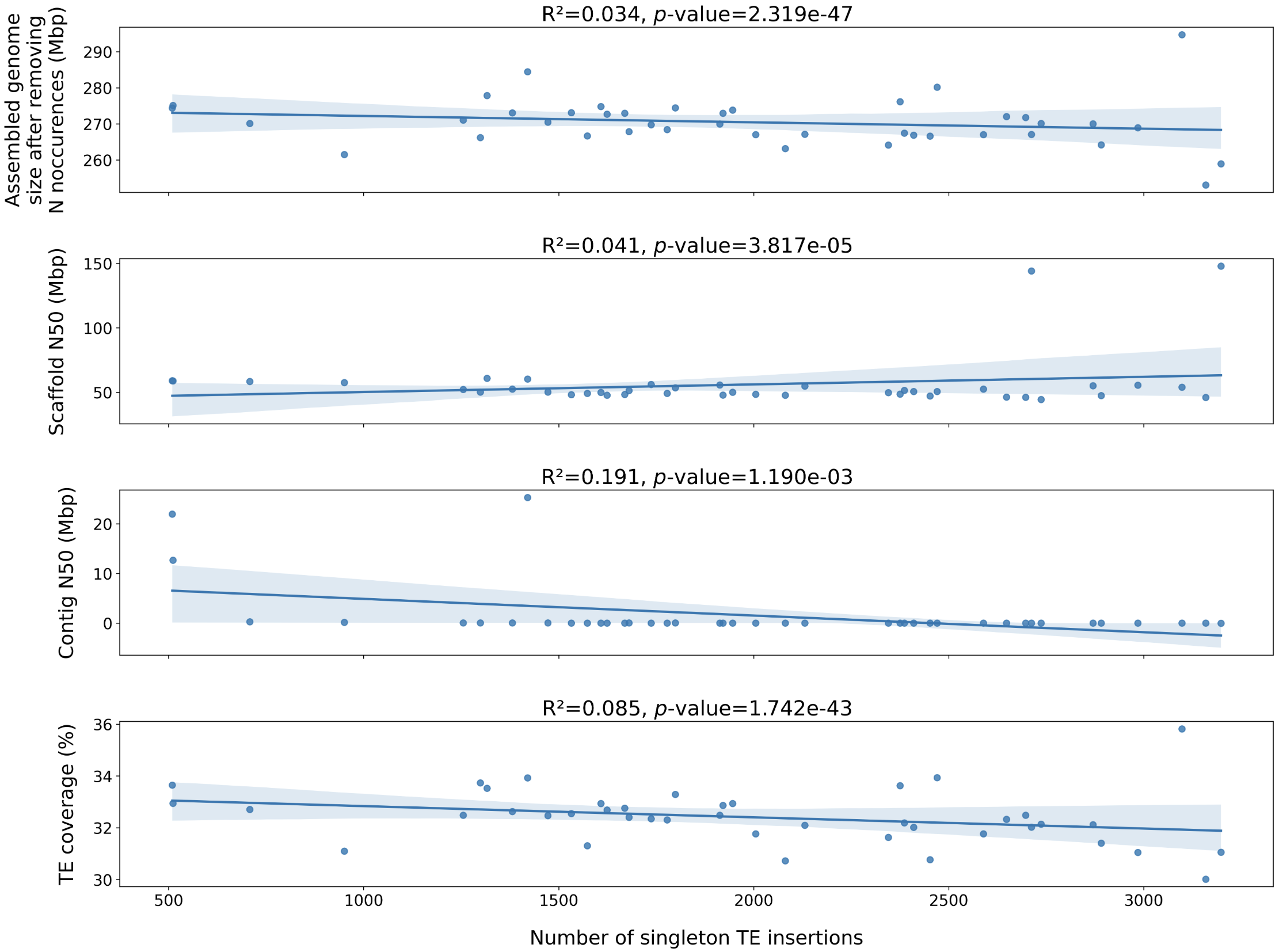


**Figure S2.** Four scatterplots comparing linear regressions for the dependent variable number of singleton TE insertions with four explanatory variables (in order): assembled genome size after removing “N” bases (R² = 0.034, *p* = 2.319e−47); scaffold N50 (R² = 0.041, *p* = 3.817e−05); contig N50 (R² = 0.191, *p* = 1.190e−03); and TE coverage (R² = 0.085, *p* = 1.742e−43). The coverage between each copy and its consensus (covcons) is set to 75–125%.

**Figure S3.** (a) TE copy lengths from panREPET (log₁₀ kbp). (b) Allele lengths from Minigraph (log₁₀ bp). (c–j) Histograms of differences between the number of accessions sharing the TE copy from panREPET and those sharing the associated allele from Minigraph. Panels (c, e, g, i) correspond to case 1, where the allele fully covers the TE copy, and panels (d, f, h, j) correspond to case 2, where the TE copy fully covers the allele. (c, d) Colors represent the proportion of accessions that are identical. (e, f) Colors represent the pangenomic compartment. (g, h) Colors represent TE copy length. (i, j) Colors represent allele length. For readability, results for covcons = 75–125% (percentage coverage of the TE copy relative to its consensus) are not shown, but they follow the same pattern.


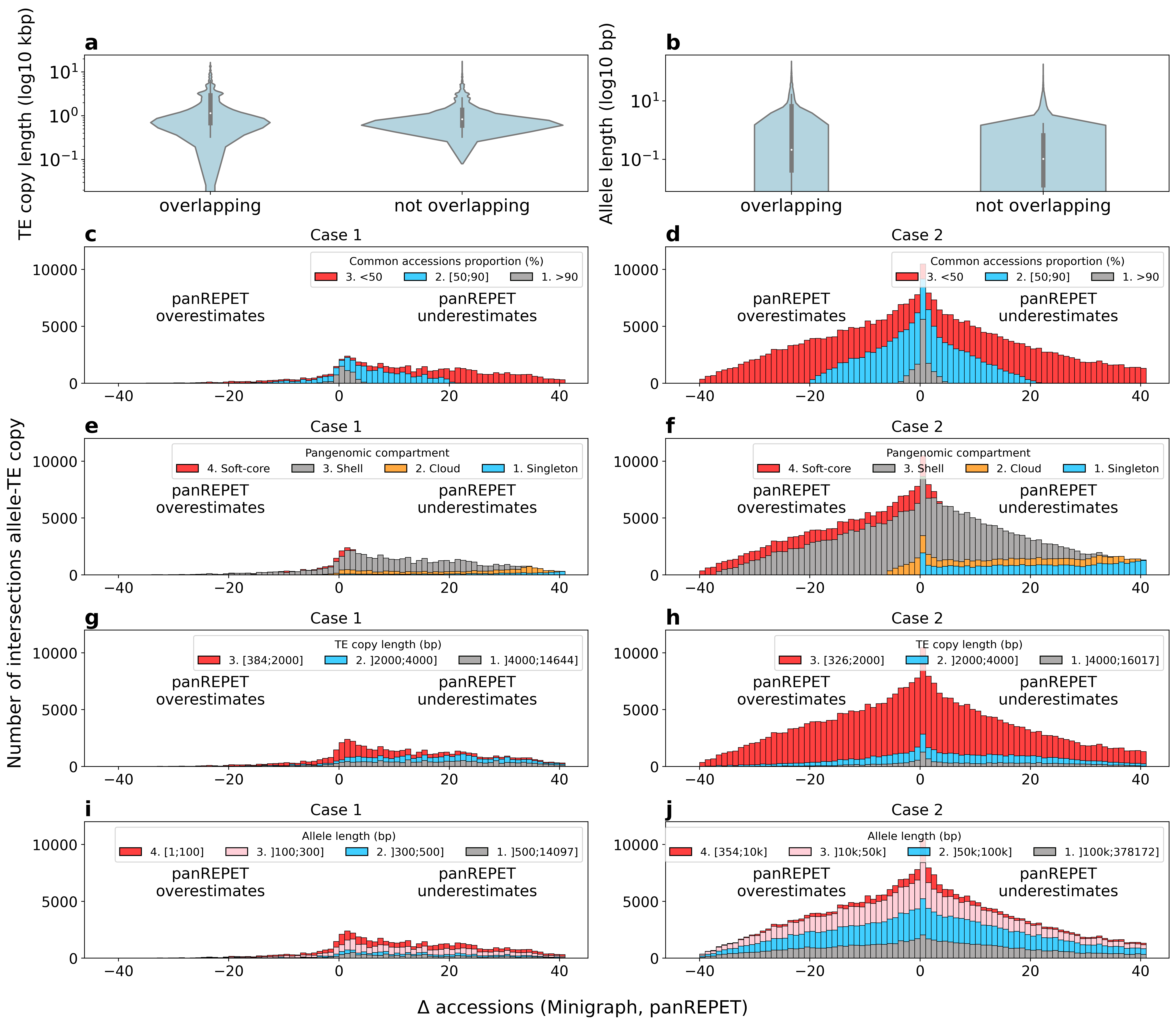

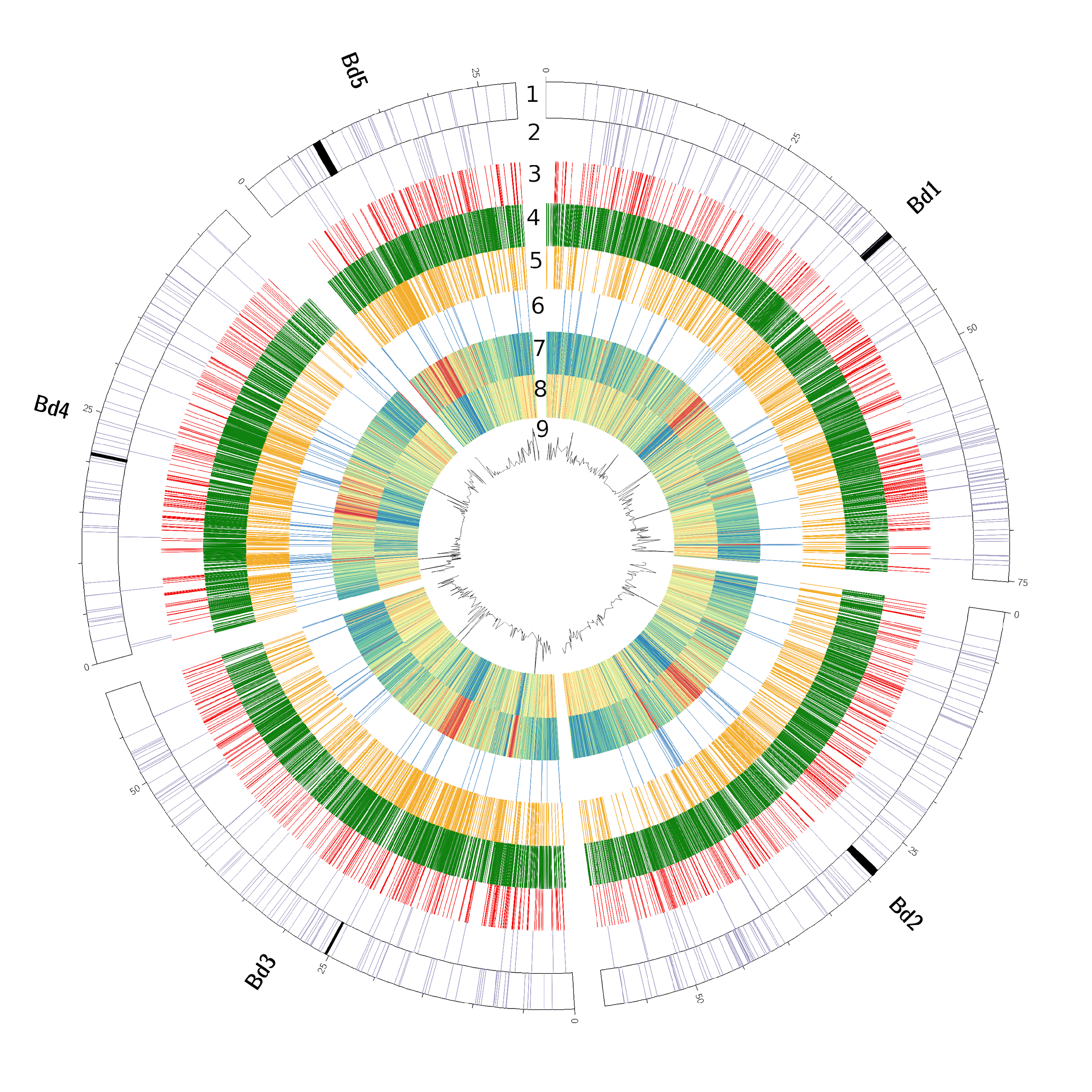


**Figure S4.** (Layers 1–6) Distribution across chromosomes of TE insertions. (Layer 1, outer layer) Core TE insertions that do not overlap genes or TFBSs; centromeres are shown in black. (Layer 2) Core TE insertions that overlap a gene or a TFBS. (Layer 3) Soft-core. (Layer 4) Shell. (Layer 5) Cloud. (Layer 6) Singleton. (Layer 7) TE density, ranging from blue to red (1–99%), calculated per 50 kbp interval. (Layer 8) Gene density, ranging from blue to red (0–84%), calculated per 50 kbp interval. (Inner layer) Recombination rates. Chromosome visualization was generated with Circos v0.69-9.


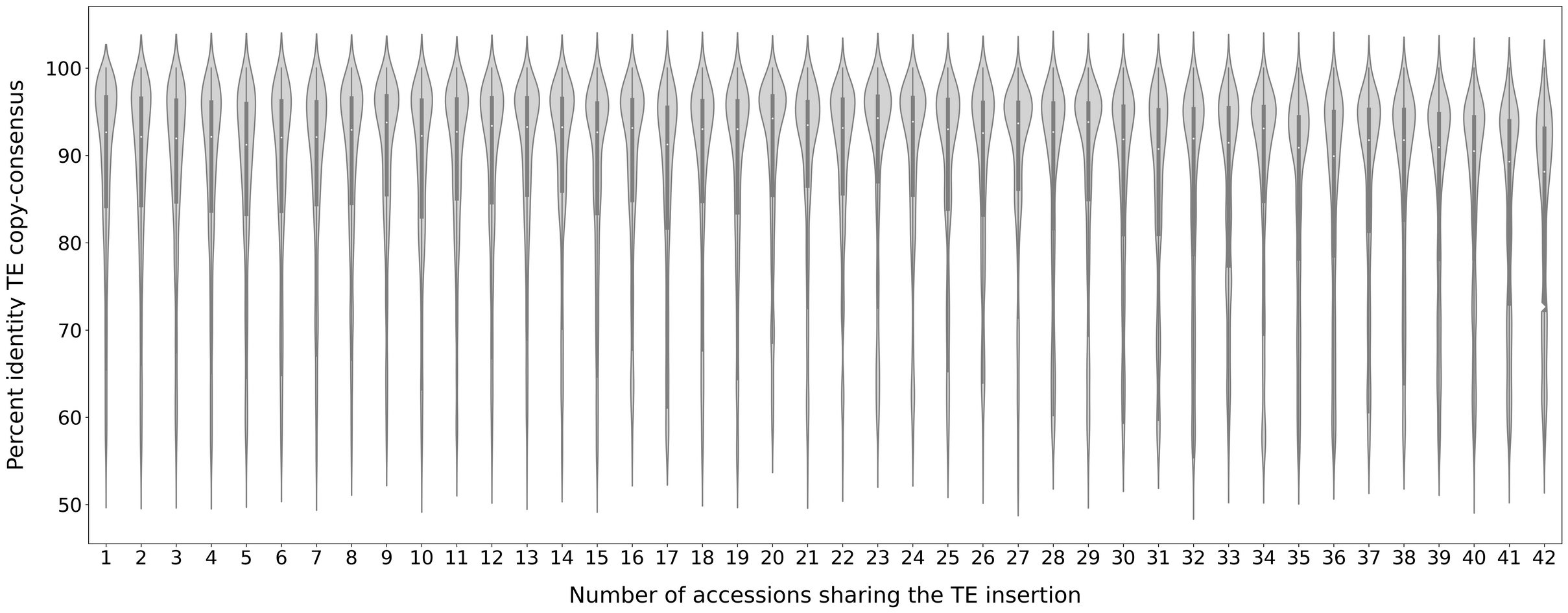


**Figure S5.** Boxplots showing how TE insertion age varies with the number of accessions sharing each insertion. Age is estimated from the percentage identity between each TE copy and its consensus.


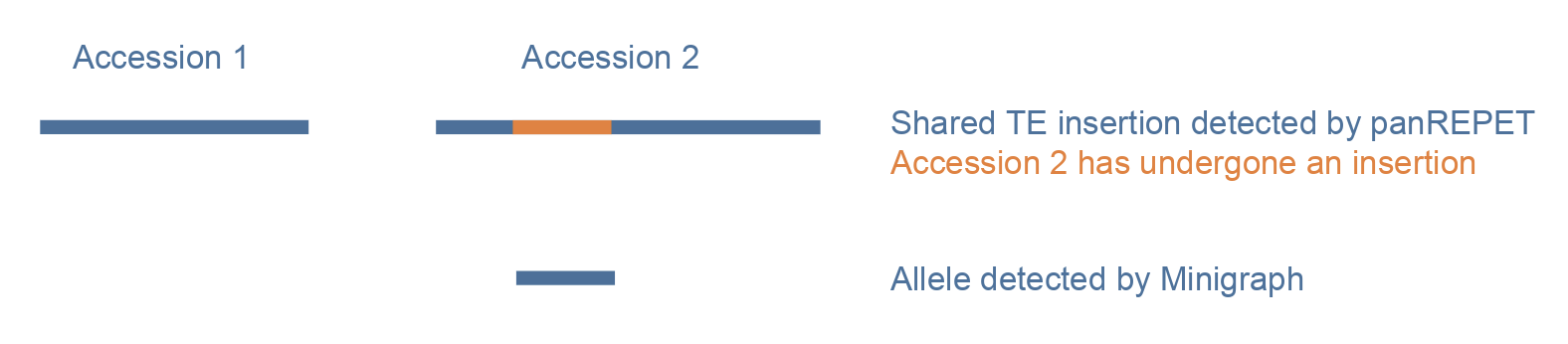


**Figure S6.** Illustration of Minigraph alleles corresponding to fragmented TE copies.
