# Supplementary material for: A reference-free pipeline for detecting shared transposable elements from pan-genomes to retrace their dynamics in a species
Source: Genome Biol. 2026 Feb 7;27:117. doi: 10.1186/s13059-026-03984-5 (PMC13059284; doi:10.1186/s13059-026-03984-5)
Supplement: Supplementary file 6 — Additional file 6. This file contains extended descriptions of the panTEannot and panREPET pipelines and benchmarking analyses. [file 13059_2026_3984_MOESM6_ESM.docx]

**Supplementary Information: Additional file 6**

**A reference-free pipeline for detecting shared transposable elements from pan-genomes to retrace their dynamics in a species**

Somia Saidi^1^, Mathieu Blaison^1^, María del Pilar Rodríguez-Ordóñez^1^, Johann Confais^1*^ & Hadi Quesneville^1^

^1^URGI - Université Paris-Saclay, INRAE, BioinfOmics, URGI, 78026, Versailles, France

*Corresponding author

Johann Confais: [johann.confais@inrae.fr](mailto:johann.confais@inrae.fr)

### Supplementary Results

###### Detailed panTEannot pipeline

We used Blaster (version 2.31) from the Singularity container te_finder_2.31.sif (<https://cloud.sylabs.io/library/hquesneville/default/te_finder>) to align chunked genomes against the consensus sequences of the TE library. The cutterDB step was configured with the parameters -l 200000 -o 10000, meaning that the genome was split into 200-kbp chunks with 10-kbp overlaps.

Matcher (version matcherThreads2.31) was executed from the Singularity container te_finder_2.31.sif (<https://cloud.sylabs.io/library/hquesneville/default/te_finder>). We used the join option (-j) to join fragments that were considered sufficiently close to represent an insertion or deletion. We also enabled the cleanup after join option (-x) to clean up fragment overlaps.

###### Detailed panREPET pipeline

*About Bidirectional best-hit detection (Step 3):*

We use Minimap2 version 2.24-r1122 with the asm20 preset, which increases sensitivity for moderately diverged sequences. The alignment scores provided by Matcher are used to identify the best hit in each pairwise comparison between every copy of one accession and all copies of the other accession.

We save computation time by comparing only TE copies annotated from the same consensus sequence. When chromosome-level assemblies are available, comparisons can also be restricted to TE copies located on the same chromosome.

A trade-off is expected between the length of the flanking region and its sequence identity. Preliminary analyses on eight *Arabidopsis thaliana* genomes (Col-0, Ler-0, KBS-Mac74, Nd-1, Ler-1, Bur-0, C24, Kro-0) showed that the larger the flanking-extension size, the greater the number of detected shared TE insertions. However, we also observed a loss of sensitivity when using larger extensions (2000 bp), likely because longer flanking regions may contain indels. For this reason, we ran the pipeline with a 500 bp flanking extension, which appeared to be the best compromise in our tests.

We also applied two filters to compare their effectiveness in reducing false positives. (i) The first filter is based on the coverage percentage of the copy plus its flanking regions with respect to its match (the sequence to which the copy was aligned). However, our preliminary analyses on the eight *Arabidopsis thaliana* genomes showed that this filter may still generate false positives when the alignment occurs mainly in the flanking extremities rather than within the copy sequence itself. (ii) We tested another filter that required each flanking extremity to be sufficiently covered by the alignment. This filter reduced the number of false positives detected when applying only the first filter. Finally, we used only a flanking-coverage filter (80% by default), which still allows the detection of partial copies with internal insertions or deletions.

###### Detailed benchmarks of panREPET

1. TEMP

Stritt *et al.* applied the TEMP tool to 53 *B. distachyon* genomes and detected 1,889 TAPs and 3,627 TIPs (Tables S2 and S3 in Stritt et al. 2018). Using the same dataset, panREPET identified 44,597 shared TE insertions (cliques). Among these, 24,085 copies are shared with the reference genome Bd21, corresponding to TAPs, while the remaining insertions are not shared with Bd21, corresponding to TIPs.

Regarding TAPs, we cannot retrieve the copy-consensus coverage from TEMP because the exact consensus is often unknown (Tables S2 and S3 in Stritt *et al.* 2018). To recover the exact consensus, we intersect TAPs detected by TEMP with the annotation performed by panTEannot and retain only the intersections sharing the same three-letter code in Wicker’s classification (Wicker *et al.* 2007). This yields 1,204 TAPs annotated by panTEannot out of the 1,889 reported by TEMP. We then filter these TAPs by keeping only those whose corresponding TE copies in Bd21 cover their reference by more than 80% according to panTEannot, which results in 396 TAPs.

2. Minigraph

Using the sequence-to-graph mapper Minigraph (Li *et al.* 2020), we detected structural variants (SVs) among the 42 *B. distachyon* genomes in graph form. In this representation, SVs are represented as bubbles, with each accession contributing one allele per bubble. Minigraph identifies 181,965 segments, 90,705 alleles, 44,163 bubbles, and an average of 3 alleles per bubble (mean and median). Alleles correspond to combinations of segments within the graph. Their lengths show substantial variation, with a mean of 5 kbp and a median of 122 bp, whereas TE copies exhibit much lower variance, with a mean of 1.7 kbp and a median of 979 bp (SF 3a–b).

We considered two cases, the first being when the allele is totally included in TE copy (i.e. the entire allele sequence covers the TE copy) (case 1) and the second when the TE copy is totally included in allele (case 2). Case 1 highlights intra-copy polymorphism (i.e. variation within the TE copies themselves) and case 2 TE copy polymorphisms (or TE insertions).

Considering only BdTR7a and the reference genome Bd21, the proportion of TE copies matching an allele is higher (53.7% and 54.8%, respectively), which is expected since the graph construction with Minigraph depends on the first introduced genomes (it increments its graph by adding genomes) (Supplementary Table 3). About the remaining 56% of TEs that do not overlap an allele from Minigraph, they exhibit a median of 831 bp and an average of 1.3 kbp, whereas TE copies overlapping an allele are slightly larger, with a median of 1.1 kbp and a mean of 2.1 kbp (SF 3a). Largest alleles overlap a TE copy with a median of 210 bp and a mean of 9.5 kbp, whereas alleles not overlapping a TE copy have a median of 100 bp and a mean of 2.8 kbp (SF 3b).

We calculated, for each TE copy–allele intersection covering more than 100% (relative to the allele in case 1, or to the TE in case 2), the difference between the number of accessions sharing the TE copy and those sharing the corresponding allele. If both numbers are identical, the difference is zero. SF 3c–j shows histograms of the distribution of these differences: values on the left indicate that panREPET underestimates, compared to Minigraph, the number of accessions sharing the TE copy, whereas values on the right indicate overestimation. We then calculated the proportion of common accessions (SF 3c–d). The closer the difference is to 0 and the proportion is to 100%, the better the shared TE insertion detected by panREPET matches the SV detected by Minigraph.

There are fewer cases where the entire allele covers the TE copy (case 1, SF 3c). In this situation, panREPET tends to underestimate the number of accessions and Minigraph to overestimate (SF 3c). This shows that, when the TE copy is incompletely detected, Minigraph loses specificity. This concerns many large TE copies (>4 kbp) (case 1, SF 3g), which correspond mostly to singleton and cloud TE copies (Fig. 2c, SF 3e). This indicates that for larger TE copies, Minigraph loses specificity (SF 6).

We observe more cases where the entire TE copy covers the allele (case 2). Here, both tools may underestimate or overestimate the number of accessions (SF 3d). This shows that Minigraph does not detect variation at the TE scale, but at the SV scale, which is broader. Adding more partial copies with panREPET (i.e. TE copies covering 75–125% of their consensus) does not change these observations.

3. GraffiTE

GraffiTE identified 47,236 SVs across all alternative accessions, comprising 111,861 TE copies classified as deletions and 48,241 classified as insertions. Among the deletions, the subset of TE copies covering their consensus between 95–105% comprised 6,487 out of the 111,861 detected across all accessions (5.7%). This subset also corresponds to 3,166 TE insertions shared with the reference genome Bd21.

On average, each SV contains approximately 2 to 3 TE copies. GraffiTE detected, on average and in the median, one accession per SV (either deletion or insertion), whereas panREPET detected a mean of 19 and a median of 18 accessions sharing a TE copy.

In terms of sequence length, GraffiTE better detected longer TE copies among those annotated by panREPET: retrieved copies have a mean length of 3.3 kbp and a median of 3.0 kbp, while non-retrieved copies have a mean of 3.2 kbp and a median of 2.4 kbp.
